# Development and Psychometric Evaluation of the Nursing Management Capacity Scale for Poststroke Dysphagia Based on the Donabedian Model

**DOI:** 10.1155/jonm/9248618

**Published:** 2026-09-20

**Authors:** Ying Zou, Yiwen Chen, Lingyi Liao, Hongyu Chen, Changyue Gao, Changyan Yuan

**Affiliations:** ^1^ Department of Rehabilitation Medicine, Daping Hospital, Army Medical University, Chongqing 400042, China, tmmu.edu.cn

**Keywords:** bifactor model, confirmatory factor analysis, Donabedian model, nurse, poststroke dysphagia, scale

## Abstract

**Background:**

Poststroke dysphagia is common and adversely affects patient outcomes, yet no standardised tool exists to assess nursing management capacity in this area.

**Aim:**

To develop and validate the nursing management capacity in poststroke dysphagia (NMC‐PSD) Scale, a self‐report instrument measuring nurses’ perception of unit‐level management capacity.

**Methods:**

The 35‐item scale was adapted from an existing checklist and refined through two rounds of Delphi consultation (I‐CVI = 0.80–1.00; S‐CVI/Ave = 0.93) and pilot testing. A cross‐sectional survey provided psychometric validation. The scale uses a mixed model: a formative structure dimension (resource inputs jointly constituting capacity) alongside reflective process and outcome dimensions. Reliability was evaluated using Cronbach’s *α* for the reflective dimensions and test–retest ICC for the total scale and the human resources composite.

**Results:**

In 410 nurses from 12 Chinese provinces, the 26‐item reflective core showed adequate internal consistency (process *α* = 0.97; outcome *α* = 0.95) and test–retest reliability (ICC = 0.98). Bifactor model fit was good: χ^2^(348) = 699.11, CFI = 0.958, TLI = 0.951, RMSEA = 0.050, SRMR = 0.027. The 26‐item reflective core showed dominant unidimensionality (ECV = 0.869, ωH = 0.963) and is recommended as the primary validated metric (range 26–130). Nine formative structural indicators, supported by content validity (S‐CVI/Ave = 0.93), are reported separately as descriptive resource inputs. Six specific factors had low independent reliability (ωHS = 0.05–0.19) and serve as conceptual or descriptive groupings only. Mean reflective total score was 54.71 (SD = 18.40); the exploratory weighted index was 207.87 (SD = 66.41).

**Conclusions:**

The NMC‐PSD Scale demonstrated acceptable psychometric properties as an individual‐level measure of nurse‐perceived unit capacity. A bifactor re‐specification, developed post hoc within the same sample, revealed a dominant general factor underlying the 26 reflective items. Independent cross‐validation is warranted. The 26‐item reflective total score is recommended as the primary validated metric; nine formative structural indicators are reported separately as descriptive resource inputs. Subscale profiles serve as conceptual or descriptive item groupings only.

**Implications for Nursing Management:**

The 26‐item reflective total score supports individual‐level inference regarding nurses’ perceptions of unit management capacity. Organisational‐level benchmarking requires further validation of within‐unit agreement and aggregation reliability. The nine structural indicators offer descriptive resource profiling, and the exploratory weighted index remains supplementary pending criterion‐related validation against objective administrative indicators.

## 1. Introduction

Stroke incidence continues to rise globally; it is the second leading cause of death worldwide and the principal contributor to mortality and disability in China [1, 2]. Moreover, the rising incidence of stroke in younger adults suggests an escalating disease burden and poses significant public health challenges [3]. Dysphagia ranks among the most prevalent complications associated with acute stroke [2]. Dysphagia is typically defined as a functional impairment of one or more swallowing organs that prevents safe and effective transport of food from the oral cavity to the stomach, causing difficulty eating [4, 5]. The incidence of dysphagia is higher after stroke than in the general population; approximately two‐thirds of stroke patients experience dysphagia [6]. Reported prevalence ranges from 8.1% to 80% [7]. Dysphagia not only directly triggers complications such as aspiration pneumonia, malnutrition and dehydration but is also closely associated with poor prognosis [8, 9]. The 30‐day mortality rate among dysphagia patients is 1.5–2 times higher than that of patients without dysphagia [10]. Repeated hospitalisations, rehabilitation and complication management increase annual direct medical costs for patients with dysphagia by US$4,510 [11] and overall healthcare costs by 40.36% [12].

Early screening and management of poststroke dysphagia may promote recovery [13]. Guidelines for the identification and management of poststroke dysphagia provide comprehensive frameworks [5, 14]. However, a cross‐sectional study in China reported that only 60.6% of nurses had completed dysphagia management training and only 36.52% consistently adhered to guideline‐based swallowing screening protocols [15]. In long‐term care settings, nursing quality influences complication rates and rehabilitation outcomes among patients with poststroke dysphagia [16, 17] and shapes their healthcare decisions [18]. In China, persistent challenges—including nonstandardised assessment processes [16], inadequate nurse training [15] and lack of proactive management [19]—have impeded evidence‐based nursing management of poststroke dysphagia [20, 21].

Existing evidence‐based practice initiatives for dysphagia care quality management focus primarily on tertiary hospitals and lack standardised evaluation criteria [18, 19], leaving a gap for standardised tools across hospital tiers. Donabedian’s three‐dimensional quality model of ‘structure‐process‐outcome’ [22] has been widely applied in clinical nursing, serving the purpose of formulating quality indicators and improving nursing outcomes [23, 24]. However, in Donabedian’s original formulation, structural measures represent the ‘presumed capacity of the provider to offer quality healthcare’ [25]; when applied at the management level, the three dimensions assess the system’s organisational readiness rather than the quality of individual care episodes. Nursing management capacity thus concerns the system‐level resources, institutional frameworks and process controls that enable consistent care delivery. It is distinct from nursing quality, which evaluates the fidelity of care behaviours [26, 27]. Validated nursing care quality scales grounded in Donabedian’s structure–process–outcome model [22] have been developed for oncology, emergency and palliative settings, yet none has been adapted to dysphagia management [28, 29]. Existing validated instruments for poststroke dysphagia predominantly capture patient‐reported symptoms or nurse‐performed screening and severity ratings at the individual patient level [30–32].

Donabedian’s framework operates as a causal‐formative model at the system level: structural inputs (personnel, equipment and systems) are heterogeneous resource inputs that jointly constitute organisational readiness rather than being interchangeable manifestations of a single latent variable [33]. In contrast, process and outcome dimensions, when operationalised as nurse‐perceived adequacy, function as reflective domains wherein items capture interchangeable perceptions of a shared underlying attribute [34].

However, in the context of nursing management capacity, process and outcome dimensions are conceptually interdependent—care processes inherently translate into patient and nurse outcomes—often leading to high empirical correlations that violate the discriminant validity assumptions of traditional second‐order factor models [22]. Recent methodological work suggests that when a scale measures a broad general construct (e.g. management capacity) while containing clinically actionable subdomains, a bifactor model is structurally preferable to a second‐order model [35, 36]. The bifactor specification allows all reflective items to load simultaneously on a general factor and orthogonal specific factors, directly quantifying the general factor’s dominance (via explained common variance [ECV]) and the reliability of the total score (via ωH), while evaluating whether specific factors possess sufficient independent reliability (ωHS) for standalone subscale interpretation [36]. This framework is particularly appropriate when high correlations between subdomains reflect a shared general core rather than construct confusion [35]. In this scale’s context, nurses likely perceive process and outcome adequacy through a ‘general capacity lens’—evaluating specific management elements based on overall organisational readiness—rendering them statistically indistinguishable yet conceptually distinct for identifying specific care gaps [37]. Therefore, adopting a bifactor model reflects this general‐dominant, specific‐supplementary measurement structure and provides quantitative justification for using the unweighted total score as the primary metric and subscales as clinical heuristics [35, 36].

To standardise dysphagia management by clinical nurses, Guo et al. [38] developed a nursing quality indicator system for poststroke dysphagia based on Donabedian’s model [22], which systematically integrates structural, process and outcome dimensions. More recently, Wan et al. [39] developed a similar indicator system for neurosurgical stroke patients (44 indicators across 11 subdomains). Both systems [38, 39] provide auditing frameworks but have not undergone psychometric validation. Although their binary response formats suffice for bedside auditing, they do not yield quantitative scores suitable for statistical comparison or trend monitoring [40], nor interval‐level metrics sensitive to subtle variations [41]. The original checklist was developed as a weighted formative index [42]; this adaptation preserves that mixed measurement logic [43] while offering a clinically intuitive framework for identifying care gaps. The present study’s incremental contribution is psychometric: it provides the first bifactor‐validated, Likert‐scaled instrument with demonstrated structural validity and reliability. Consequently, a psychometrically robust self‐report instrument for measuring nurse‐perceived unit nursing management capacity in poststroke dysphagia (NMC‐PSD) care remains unavailable. By converting the checklist into a Likert‐scaled instrument with demonstrated reliability and structural validity under a bifactor framework, this study extends the existing repertoire of Donabedian‐based nursing quality measures to poststroke dysphagia care.

In Chinese hospitals, fragmented electronic documentation systems often preclude comprehensive objective auditing across all quality indicators [44]. Against this backdrop, nurses’ subjective perceptions constitute a measurement dimension with established convergent validity and offer actionable insights for staffing‐related decision‐making [45, 46]. Self‐assessment approaches further enable rapid, cost‐effective internal auditing supporting iterative quality improvement [47, 48]. Cross‐unit benchmarking would require evidence of within‐unit agreement and aggregation reliability, which are not available in this study. As a research instrument, the scale generates interval‐level metrics suitable for pre–post intervention evaluation and multisite comparison.

To address this gap, we adapted the original checklist [38] into a Likert‐scaled instrument: the NMC‐PSD Scale. This scale measures nurses’ perceptions of nursing management capacity in their own clinical units. Items use a unit referent, but each score reflects an individual perception. Without within‐unit agreement and aggregation reliability, unit‐level inference is not supported [49]. The scale is completed by registered nurses currently practising in stroke or rehabilitation units where poststroke dysphagia patients are routinely managed. It is neither a test of individual competence nor a comprehensive hospital audit, but a unit‐focused internal assessment. The specific objectives were as follows: (1) to adapt the binary indicators from Guo et al.’s system into a multipoint Likert scale; (2) to evaluate the psychometric properties of the NMC‐PSD Scale, including reliability and structural validity; and (3) to describe current NMC‐PSD scores in a sample of nurses from Chinese stroke units, thereby establishing baseline data for future quality improvement interventions.

## 2. Methods

This study adapted assessment indicators for care quality among patients with dysphagia in China into a five‐point Likert scale. This was a cross‐sectional, multiphase scale adaptation and validation study. The study comprised three sequential phases: (1) item generation, (2) content validation and (3) psychometric validation. The NMC‐PSD Scale was developed between December 2024 and February 2025. A cross‐sectional survey was conducted between February and April 2025 to examine the scale’s reliability and validity. Nurses were recruited using convenience sampling from hospitals across 12 Chinese provinces.

### 2.1. Item Generation

The scale was developed through three sequential steps: (1) item operationalisation and adaptation, (2) expert consultation and (3) pilot testing. Permission to adapt the original checklist into a self‐report Likert scale was granted by the developers of the source instrument [38].

#### 2.1.1. Source Instrument and Theoretical Framework

Guo et al. [38] developed nursing quality indicators for poststroke dysphagia through a rigorous Delphi expert consultation process. In their study, expert authority coefficients were 0.958 (Round 1) and 0.964 (Round 2), and Kendall’s W coefficients were 0.216 and 0.340 respectively (*p* < 0.01). The analytic hierarchy process was used to determine hierarchical weights, with consistency ratios < 0.1 indicating validity [50]. Because the original checklist did not assign explicit scores to tertiary indicators, their weights were established through the expert consultation process. The present study retained this hierarchical classification and weighting system [51].

#### 2.1.2. Item Operationalisation and Adaptation

The original checklist’s 35 structure–process–outcome indicators were converted into self‐report items through one‐to‐one deductive operationalisation. Objective indicators (e.g. physician‐to‐bed ratio, equipment availability) were transformed into statements about nurse‐perceived adequacy, rated on a 5‐point Likert scale. Wording decisions preserved the original semantic domain by converting objective structural metrics into perceptual statements, thereby ensuring that respondents could rate adequacy without calculating or verifying objective metrics. All 35 indicators were retained because the core panel (comprising 3 nursing management researchers with doctoral, master’s and bachelor’s degrees, respectively, and 2 clinical specialists with master’s and bachelor’s degrees respectively) confirmed that none was redundant or nontransformable. Item construction followed five a priori criteria: unidimensionality, perceptual measurability, semantic clarity, framework fidelity and scoring compatibility [51]. Accordingly, all 35 items proceeded directly to the two‐round Delphi consultation. The original dimensional framework was retained, with primary, secondary and tertiary indicators established. Two researchers independently adapted indicators into quantifiable statements using a 5‐point Likert scale (1 = strongly disagree; 5 = strongly agree). Discrepancies between the two researchers were resolved by consensus within the core panel; persistent disagreements were referred to the original checklist developers for arbitration. This process formed the initial item pool. The research team verified the adapted scale with the original developers to preserve measurement objectives. During this verification, contextual applicability for Chinese public hospitals was explicitly assessed; all items were subsequently evaluated by the expert panel for relevance within the local public hospital setting, confirming that no item required deletion for cultural inappropriateness. For subsequent psychometric analyses, raw unweighted Likert scores were used [51, 52]. Hierarchical weights from the source checklist were not applied in the measurement model; they were retained solely for generating the final clinically interpretable composite score [51].

#### 2.1.3. Weighted Composite Scoring (Exploratory)

The final scoring system was derived from the Delphi consensus weights through a standardised three‐stage conversion [53].

• Stage 1: Weight percentage computation. Let *w*_*i*_ denote the raw Delphi‐AHP weight for item *i* (*i* = 1, 2, …, 35), obtained by multiplying the three‐level hierarchical weights (dimension × subdimension × item). Let $W=\sum_{i=1}^{35}w_{i}$, denote the sum of all raw weights. The weight percentage for item *i* is computed as:
(1) Pi=wiW×100100, where ∑i=135Pi=.
• Stage 2: Item‐level weighted score assignment. Let *k* ∈ {1, 2, 3, 4, 5} denote the Likert response option, with standardised proportional coefficients *c*_*k*_ defined as:
(2) c5=1.00.80.60.40.2,c4=,c3=,c2=,c1=.
 The weighted score for item *i* at response *k* is given by
(3) Si,k=roundPi×5×ck.
 where round (·) denotes rounding to the nearest integer. The base score for the ‘Strongly Agree’ option (*k* = 5) is *B*_*i*_ = *P*_*i*_ × 5, and all other options are derived by multiplying this base score by the corresponding coefficient and rounding.
• Stage 3: Composite score computation. For a respondent with Likert responses *r*_*i*_ ∈ {1, 2, 3, 4, 5} for each item *i*, the total weighted composite score is calculated as:
(4) Total=∑i=135Si,ri=∑i=135roundPi×5×cri

Theoretical bounds. Because ∑*P*_*i*_ = 100 exactly, the unrounded theoretical maximum is $\sum_{i=1}^{35}\left(P_{i}\times 5\times c_{5}\right)=5100500\times =$. After item‐level rounding, the maximum possible total score remains exactly 500 because the positive and negative rounding errors cancel out (net rounding error = 0). The minimum possible total score (all items = 1) is 95, because *S*_*i*,1_ = round(*P*_*i*_ × 5 × 0.2) = round(*P*_*i*_), and the net rounding error across all 35 items is −5. The complete item‐level scores for all response options are provided in Supporting File 1, Table S2b, and the full calculation workbook is available in Supporting File 1, Table S1.

### 2.2. Content Validation

#### 2.2.1. Expert Panel and Delphi Consultation

Ten healthcare experts—two nursing administrators, two clinical nurses, three clinical physicians and three rehabilitation therapists—were recruited by purposive sampling. Each expert met the following selection criteria: either ≥ 15 years of healthcare experience or ≥ 5 years of specialised dysphagia management expertise; a senior professional title (associate professor or above in academia, or deputy director‐level or above in clinical practice); and a bachelor’s degree or higher. Experts who participated in the preliminary item generation core panel were excluded to prevent bias.

The same panel completed two anonymous electronic consultation rounds (Round 1: January 2025; Round 2: February 2025) [54], blinded to one another’s identities and ratings, with no discussion permitted. The response rate was 100% in both rounds, and the mean expert authority coefficient (Cr) was 0.88 (range 0.70–0.95). In Round 1, experts rated item importance on a 4‐point scale and provided written comments, which were collated anonymously and returned to the panel with the revised questionnaire. In Round 2, experts rerated item relevance and clarity (Section 2.2.2). Consensus was defined a priori as meeting the content‐validity thresholds described in Section 2.2.2, with an item‐level coefficient of variation (CV) < 0.25 for all items; all criteria were met in Round 2, and the consultation was terminated. The full procedure, round‐specific changes and agreement statistics are provided in Supporting File 3.

#### 2.2.2. Content Validity Assessment

Experts assessed item relevance using a 4‐point ordinal scale (1 = irrelevant, 2 = somewhat relevant, 3 = quite relevant, 4 = highly relevant). Ratings of 3 or 4 were dichotomised as ‘relevant’. The item‐level content validity index (I‐CVI) and the scale‐level content validity index average (S‐CVI/Ave) were used to evaluate content validity. An I‐CVI ≥ 0.78 and an S‐CVI/Ave ≥ 0.90 indicate adequate content validity [55]. Additionally, Polit–Beck modified kappa (*k* ^∗^) was calculated for each item to adjust for chance agreement, with *k* ^∗^ > 0.74 indicating excellent agreement beyond chance [56]. Experts also evaluated content clarity and linguistic precision for target users, providing revision suggestions for ambiguous entries [57].

#### 2.2.3. Pilot Testing

Fifteen experienced clinical nurses completed the scale in a pilot test to provide feedback regarding clarity and comprehensibility [58].

### 2.3. Psychometric Validation

#### 2.3.1. Participants and Setting

The target population included nurses who met the following criteria: (1) held nursing qualifications with a valid registered nurse licensure; and (2) had ≥ 1 year of clinical experience and were authorised to perform independent assessments after completing departmental training. Nurses were excluded if they were uninvolved in dysphagia care, unfamiliar with specialised assessment instruments or had health conditions impairing survey participation (e.g. sensory or communication deficits).

Because the scale was adapted from an instrument with a theoretically predefined factor structure, confirmatory factor analysis (CFA) was used to verify the retained dimensionality without prior exploratory factor analysis [59]. A minimum sample size of 200 is recommended for CFA [60], and psychometric conventions recommend five to ten participants per item [61, 62]. With 35 items in the formal NMC‐PSD Scale, this yielded a target sample size of 175–350 participants. We therefore adopted 350 valid responses as the minimum threshold for initiating psychometric analyses. Data were collected via an online survey platform with real‐time response monitoring. Data collection remained open until a natural closure point was reached across all participating centres, yielding a final sample of 410 valid questionnaires (11.7 participants per item) and satisfying both criteria. A total of 41 participants (10.0% of the initial sample) were invited to complete the retest two weeks after the initial survey; all 41 completed it (response rate 100%). Test–retest reliability was assessed using a two‐way random‐effects, absolute‐agreement, single‐measures ICC(2,1) model [63]. The survey was distributed as a single anonymous link through hospital nursing departments in 12 provinces. To protect respondents evaluating their own units, hospital and unit identifiers were not recorded; therefore, the data support only individual‐level analyses. Potential clustering was addressed through sensitivity analyses (Sections 2.3.3 and 2.3.5). All psychometric analyses, including CFA, reliability testing and validity assessments, were conducted using unweighted raw 5‐point Likert‐scale scores [51, 52]. The original checklist weights were not incorporated into the measurement model. The weighted scores, derived from the original Delphi‐AHP weights, are calculated separately as an exploratory descriptive index and are not subject to the psychometric validation procedures described herein.

#### 2.3.2. Data Collection

Electronic questionnaires were distributed to nurses in hospitals across 12 Chinese provinces spanning eastern, southern, western and northern regions. Hospital nursing departments shared survey links to recruit participants. The questionnaire comprised: (1) sociodemographic and work variables (age, gender, marital status, education level, clinical ladder rank, nursing experience and hospital level); and (2) the adapted NMC‐PSD Scale. Completed surveys were transmitted directly to the research team via secure servers. Participants received study purpose disclosures and provided informed consent prior to survey access. To ensure data quality, the survey platform restricted each account to a single submission, thereby preventing duplicate entries. After data export, straight‐line responses (i.e., identical answers across all items) were manually identified and excluded as careless responses [64]. This anonymous survey implemented standardised data anonymisation protocols to ensure objective evaluation free from bias. Participating units and hospitals were blinded to individual‐level results, preventing potential conflicts of interest from compromising assessment integrity.

#### 2.3.3. Statistical Analysis

Data were analysed using IBM SPSS Statistics 26.0 (SPSS Inc., Chicago, IL, USA). The statistical significance threshold was set at *α* < 0.05. Continuous data that followed a normal distribution (Shapiro–Wilk *p* > 0.05) were reported as mean ± standard deviation (SD), whereas skewed variables were reported as median and interquartile range (IQR). Categorical variables were reported using frequencies and percentages (%). To evaluate clustering effects, intraclass correlation coefficient (ICC(1)) values and design effects were computed from random‐intercept mixed models at the two available grouping levels (province and city), with an analysis of variance (ANOVA) estimator as a sensitivity analysis. Because unit‐level clustering was unobservable, an assumption‐based scenario analysis (ICC = 0.05–0.15; average cluster size = 10) was added as a conservative robustness check, not as cluster‐adjusted estimation.

#### 2.3.4. Measurement Model Specification

Given the NMC‐PSD’s origin as a weighted indicator checklist grounded in Donabedian’s causal framework, we explicitly specify a mixed measurement model [34, 43].

Structure dimension (formative): At the second‐order level, the structural dimension was conceptualised as a formative construct composed of three causal indicators—human resources, facilities and equipment and management systems. These subdimensions represent heterogeneous, noninterchangeable resource inputs that jointly constitute nursing management capacity; they are not expected to be caused by a single latent structural capacity variable or to co‐vary with one another [33, 42]. Consequently, internal consistency metrics (e.g. Cronbach’s *α*) and convergent validity metrics (e.g. composite reliability [CR], average variance extracted [AVE]) are not appropriate or reported for the structural dimension as a whole. Instead, validity is established through expert consensus content validity (I‐CVI, S‐CVI), a priori hierarchical weights and the theoretical necessity of each component [42]. Because the five human resources items likewise represent noninterchangeable resource inputs—nurse‐to‐bed ratio, physician‐to‐bed ratio, nurse allocation by level, multidisciplinary team functioning and therapist‐to‐bed ratio—that are not expected to be caused by a single latent factor or to co‐vary with one another, the human resources subdimension (1.1), like facilities and equipment (1.2) and management systems (1.3), was specified as an observed composite indicator directly linked to the second‐order formative structural dimension [33, 42, 43].

Process and outcome dimensions (reflective): The process and outcome dimensions are conceptualised as reflective second‐order constructs. Their subdimensions (e.g. nursing assessment, nursing practice, nurse‐related outcomes, patient‐related outcomes) are treated as reflective indicators of higher‐order latent variables representing ‘perceived process adequacy’ and ‘perceived outcome quality’ respectively. Items within each subdimension are expected to be interchangeable manifestations of their respective latent constructs, justifying the use of Cronbach’s *α*, CFA, CR and AVE for these domains [34].

This mixed specification aligns with the theoretical distinction between system‐level resource inputs (structure, formative) and clinical practice perceptions (process/outcome, reflective) [65].

#### 2.3.5. Confirmatory Factor Analysis and Structural Validity

Given that the original indicators were theoretically grounded, confirmatory factor analysis was performed. An initial second‐order CFA with formative structural composites was specified and estimated in IBM SPSS AMOS 24.0 (IBM Corporation, Armonk, NY, USA) using maximum likelihood estimation with Bollen–Stine bootstrap (2,000 samples) [66]. The Bollen–Stine bootstrap *p* value for this rejected second‐order model was 0.004; however, because the specification was abandoned due to the discriminant validity failures noted above, inferential statistics for the adopted bifactor model rely on maximum likelihood robust (MLR) standard errors rather than bootstrap resampling. This model exhibited intractable discriminant validity failures (second‐order heterotrait–monotrait ratio [HTMT] = 1.06; first‐order HTMT > 0.90 for several pairs), indicating that the process and outcome dimensions were not empirically distinct [67]. A supplementary principal component analysis (PCA) of the 26 reflective items was conducted as a post hoc dimensionality diagnostic: the first unrotated component extracted > 40% of variance, with all item loadings > 0.40, suggesting a dominant general factor underlying the reflective items. Combined with the HTMT failures, this pattern indicated that the process and outcome subdimensions share a strong general core rather than constituting distinct reflective constructs—a structural condition for which a bifactor specification is statistically and conceptually preferable to a second‐order model [35, 36]. Because the bifactor respecification was developed in the same sample used for estimation, the resulting fit and indices require confirmation through independent cross‐validation.

**FIGURE 1:**
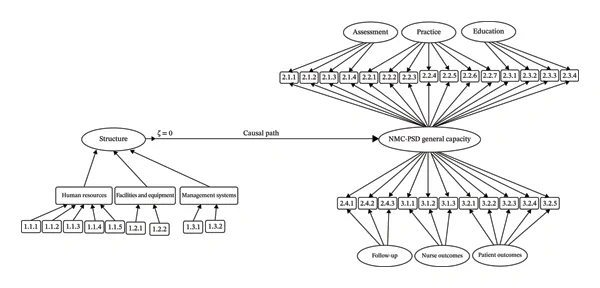
Bifactor model path diagram for the NMC‐PSD Scale.

Consequently, a bifactor CFA was adopted as the primary model and estimated in R (Version 4.6.0) using the lavaan package (Version 0.6–19) [68]. Model parameters were estimated using MLR with Satorra–Bentler scaled test statistics [69]. The fitted model was a formative–bifactor hybrid (Figure 1): the three formative composites of the structural dimension (human resources, facilities and equipment and management systems) were specified as observed exogenous single‐indicator composites and entered as exogenous predictors of the general factor, whereas the 26 reflective process and outcome items simultaneously loaded on the general factor and on six orthogonal specific factors (assessment, practice, education, follow‐up, nurse outcomes and patient outcomes). Orthogonality was enforced by fixing all covariances between the general factor and each specific factor, and among all specific factor pairs, to zero (e.g. gen ∼∼ 0∗assess + 0∗practice + …; assess ∼∼ 0∗practice + …).

Bollen–Stine bootstrap (2,000 samples) was additionally requested to obtain an adjusted chi‐square test statistic and bootstrap standard errors. However, the orthogonal bifactor resampling produced pervasive convergence failures and Heywood cases in the bootstrap distribution, rendering bootstrap standard errors and percentile confidence intervals uninterpretable. Consequently, all inferential statistics for factor loadings—robust standard errors, *z* values and 95% Wald confidence intervals—are based on MLR (maximum likelihood with robust standard errors; Satorra–Bentler scaled test statistics). MLR provides standard errors robust to multivariate non‐normality without resampling and was adopted after the Bollen–Stine bootstrap failed for the orthogonal bifactor model. MLR and bootstrap standard errors are distinct methods; no claim of equivalence is made [70]. Model fit was evaluated using MLR‐scaled *χ* ^2^, comparative fit index (CFI), Tucker–Lewis index (TLI), root mean square error of approximation (RMSEA) (90% CI) and standardised root mean square residual (SRMR). Cut‐off criteria were as follows: CFI/TLI ≥ 0.95 (excellent) or ≥ 0.90 (acceptable); RMSEA ≤ 0.08; SRMR ≤ 0.08 [58, 70]. The bifactor model was compared against the rejected second‐order CFA as a benchmark.

This specification preserves Donabedian’s causal chain [22], in which structural inputs enable process and outcome capacity, while accommodating the dominant unidimensionality observed in the reflective items. The formative–reflective distinction follows Jarvis et al. [34] and Becker et al. [43]; the bifactor partition follows Reise [35] and Rodríguez et al. [36]. The nine individual structural items did not load on the general factor; only their composite scores entered the structural regression.

#### 2.3.6. Construct Validity and the Bifactor Indices

Construct validity under the bifactor framework was evaluated via three modern indices [36]: (i) explained common variance (ECV), the proportion of common variance attributable to the General Factor, with ECV ≥ 0.70 indicating essential unidimensionality; (ii) hierarchical omega for the general factor (ωH), with ωH ≥ 0.80 indicating reliable interpretation of the total score; and (iii) hierarchical omega for the specific factors (ωHS), with ωHS ≥ 0.50 indicating reliable subscale interpretation. ECV, ωH and ωHS were computed using the bifactorIndices function from the BifactorIndicesCalculator package (Version 0.2.2) in R (Version 4.6.0) [36, 71]. Complete bifactor standardised loadings are reported in Supporting File 2, Table S4, and the reproducible lavaan model syntax and full R output are provided in Supporting File 4.

#### 2.3.7. Item Retention Protocol

In Stage 1, for items within reflective subdimensions, psychometric screening was conducted: items were flagged if their CITC was below 0.30 or their standardised factor loading was below 0.40 [72, 73]. Internal consistency was evaluated using Cronbach’s *α* for reflective subdimensions and dimensions; Cronbach’s *α* ≥ 0.7 were deemed acceptable [74]. Formative structural indicators (all items within human resources, facilities and equipment and management systems) were exempt from these reflective‐scale criteria because interitem covariance is not theoretically expected under formative specification [34, 42]; their retention was based on content validity (I‐CVI ≥ 0.78) and theoretical necessity. In Stage 2, content validity was verified via two rounds of Delphi panel review (*n* = 10) using the I‐CVI, S‐CVI/Ave and modified kappa thresholds specified in Section 2.2.2. Interexpert consistency was additionally assessed using the ICC under a two‐way random‐effects model with absolute agreement; an ICC ≥ 0.75 indicated good inter‐rater reliability [63]. To account for chance agreement, Polit–Beck modified kappa (*κ* ^∗^) was computed for each item; *κ* ^∗^ ≥ 0.74 was considered excellent agreement beyond chance [56]. In Stage 3, items passing Stage 2 were adjudicated for theoretical necessity and structural irreplaceability. In this study, no items were removed, as none failed all three stages.

### 2.4. Ethical Considerations

The research protocol was approved by the Biomedical Ethics Committee of the Army Medical Center of the PLA (Daping Hospital) (approval number 2023‐277). This approval covered the overall study protocol, including anonymous survey recruitment across multiple provinces. The survey was distributed as a single anonymous link through hospital nursing departments, which acted solely as passive conduits for link dissemination; no hospital functioned as a formal research site or data collection centre. Participation was voluntary and anonymous. No hospital or unit identifiers were collected. Participants’ geographic distribution (12 provinces) was captured through self‐report demographic items only; the research team cannot identify specific participating institutions. As the study was anonymous survey research with no intervention and minimal risk, the reviewing committee explicitly determined that separate local ethics approvals were not required. All procedures followed were in accordance with the ethical standards of the 1964 Helsinki Declaration and its later amendments. Informed consent was obtained from all participants prior to their inclusion in the study.

### 2.5. AI‐Generated Content Declaration

This article was independently authored by the authors. During the writing process, the Kimi AI tool was used to polish language, optimise logical flow and refine expressions throughout the text. The core viewpoints, content, ideas and conception of this article are original to the authors. The AI only provides auxiliary optimisation and does not participate in core creation.

## 3. Results

### 3.1. Characteristics of the Participants

The study recruited 414 nurses from 12 provinces in China. Respondents were located predominantly in Chongqing, southwestern China. After excluding two questionnaires with identical responses across all items and two refusals to participate, 410 valid responses were retained. As shown in Table 1, participants’ mean age was 34.14 years (SD 6.95; range 21–55), and 93.90% were female. Nearly three‐quarters were married (*n* = 297, 72.44%), and most held bachelor’s degrees (*n* = 357, 87.07%). The most common clinical ladder ranks were N2 (*n* = 141, 34.39%) and N3 (*n* = 145, 35.37%). The majority of participants had 11–20 years of work experience (*n* = 172, 41.95%). The majority of data were collected from tertiary hospitals (*n* = 295, 71.95%). The retest subsample (*n* = 41) was demographically comparable to the full sample (Table 1), with a mean age of 34.20 years (SD 6.24) and similar distributions of education and clinical ladder rank.

**TABLE 1:** Sociodemographic and work characteristics of participants (*N* = 410).

Variables	Characteristic	*N*	%	Mean ± SD
Age				34.14 ± 6.95

Gender	Female	385	93.90	
	Male	25	6.10	

Marital status	Single	106	25.85	
	Married	297	72.44	
	Divorced or widowed	7	1.71	

Education level	University diploma	9	2.20	
	Bachelor’s degree	357	87.07	
	Master’s degree and above	44	10.73	

Clinical ladder rank	N0‐N1	88	21.46	
	N2	141	34.39	
	N3	145	35.37	
	N4 or above	36	8.78	

Years of experience	1–5 years	96	23.41	
	6–10 years	92	22.44	
	11–20 years	172	41.95	
	> 20 years	50	12.20	

Hospital level	Tertiary hospital	295	71.95	
	Secondary hospital	115	28.05	

### 3.2. Scale Construction

#### 3.2.1. Content Validity

The NMC‐PSD Scale demonstrated adequate content validity, with an S‐CVI/Ave of 0.93 (Table 2). All items achieved I‐CVI scores of at least 0.78 (range 0.80–1.00), and modified kappa values ranged from 0.79 to 1.00 (mean 0.93) (Supporting File 3, Table S6c). Although all items met the I‐CVI threshold for content relevance, this did not preclude psychometric scrutiny of the reflective items. The ten‐rater ICC(2,10) was 0.684 (single‐rater ICC = 0.178) in Round 1, but ceiling effects in Round 2 reduced variance, rendering ICC (and W) nondiscriminatory. Kendall’s W was computed for both rounds: Round 1, *W* = 0.257 (χ^2^ = 87.30, df = 34, *p* < 0.001); Round 2, *W* = 0.128 (χ^2^ = 43.42, df = 34, *p* = 0.129). Please refer to Supporting File 3 for the specific report of the two expert consultations.

**TABLE 2:** Results of item analysis, reliability and content validity for the NMC‐PSD Scale (*N* = 410).

Items	I‐CVI	CITC	Cronbach’s *α*	CID
Dimension 1			—	
1.1		—	—	
1.1.1	1.00	—		—
1.1.2	1.00	—		—
1.1.3	1.00	—		—
1.1.4	1.00	—		—
1.1.5	1.00	—		—
1.2		—	—	
1.2.1	1.00	—		—
1.2.2	0.90	—		—
1.3		—	—	
1.3.1	0.90	—		—
1.3.2	0.80	—		—
Dimension 2		0.97	0.97	
2.1		0.87	0.89	
2.1.1	0.90	0.75		0.96
2.1.2	0.90	0.71		0.96
2.1.3	1.00	0.75		0.96
2.1.4	1.00	0.80		0.96
2.2		0.89	0.96	
2.2.1	1.00	0.77		0.96
2.2.2	0.90	0.78		0.96
2.2.3	1.00	0.80		0.96
2.2.4	0.80	0.82		0.96
2.2.5	1.00	0.83		0.96
2.2.6	0.80	0.80		0.96
2.2.7	0.90	0.86		0.96
2.3		0.91	0.93	
2.3.1	0.90	0.82		0.96
2.3.2	0.90	0.82		0.96
2.3.3	1.00	0.85		0.96
2.3.4	1.00	0.84		0.96
2.4		0.90	0.92	
2.4.1	0.90	0.84		0.96
2.4.2	0.90	0.86		0.96
2.4.3	0.90	0.82		0.96
Dimension 3		0.94	0.95	
3.1		0.91	0.94	
3.1.1	0.80	0.86		0.96
3.1.2	1.00	0.88		0.96
3.1.3	0.90	0.87		0.96
3.2		0.94	0.94	
3.2.1	0.80	0.79		0.96
3.2.2	0.90	0.84		0.96
3.2.3	0.90	0.85		0.96
3.2.4	1.00	0.78		0.96
3.2.5	0.90	0.86		0.96
S‐CVI/Ave	**0.93**			
Total			**0.96**	

*Note:* CID, Cronbach’s *α* if item deleted; S‐CVI/Ave, average scale content validity index. The structural dimension is specified as a formative second‐order construct; its subdimensions facilities and equipment (1.2) and management systems (1.3) were treated as observed formative indicators directly linked to the second‐order structural dimension. Consequently, CITC and CID are not reported for these formative indicators, and Cronbach’s *α* is not reported at the Structural Dimension level. The total Cronbach’s *α* (0.96) is reported descriptively but is not interpreted as evidence of unidimensionality across the entire scale. The structural dimension and all three of its subdimensions (human resources 1.1, facilities and equipment 1.2, management systems 1.3) are specified as formative; CITC, CID and Cronbach’s *α* are derived from reflective assumptions and are therefore not reported for structural items. Item‐level VIFs within the human resources composite ranged from 1.32 to 2.00, confirming that the five items capture nonoverlapping resource facets. Bold values indicate the overall content validity and reliability of the scale.

Abbreviations: CITC, corrected item‐total correlation; I‐CVI, item content validity index.

#### 3.2.2. Pilot Testing and Item Refinement

During pilot testing, the scale was completed in a median time of 3.39 min (IQR 2.16–4.41). Expert reviewers confirmed the scale’s overall comprehensibility but recommended greater specificity for two items to improve clinical utility. Consequently, item 2.2.1 was refined from ‘Able to select appropriate feeding routes’ to ‘Able to select appropriate feeding routes (e.g. oral intake, intermittent oro‐oesophageal tube feeding, nasogastric tube feeding)’, and item 3.2.1 was revised from ‘Near‐absence of dysphagia‐related adverse events’ to ‘Near‐absence of dysphagia‐related adverse events (as documented in electronic health records)’. These modifications provide explicit operational definitions and strengthen the instrument’s clinical applicability. Following expert consensus and pilot testing refinements to items and instructions, the finalised scale comprised three core dimensions and 35 items. Following scale weighting procedures (Supporting File 1, Table S1), the NMC‐PSD Scale was created (Supporting File 1, Table S2a for the Likert items, Table S2b for the weighted scoring).

#### 3.2.3. Item Analysis

For the 26 items within the reflective process and outcome subdimensions, corrected item‐total correlations ranged from 0.71 to 0.88, all exceeding the 0.30 threshold (Table 3). The nine structural items (1.1.1–1.3.2) are formative indicators; CITC, standardised loadings and internal‐consistency criteria are derived from reflective measurement assumptions and are therefore not applicable to them [34, 42]. Their retention rested on content validity (all I‐CVI ≥ 0.80; modified kappa 0.79–1.00) and on the theoretical necessity of each resource input within Donabedian’s structure component [42].

**TABLE 3:** Goodness‐of‐fit statistics for alternative measurement models (*N* = 410).

Model	Type	*χ* ^2^	df	CFI	TLI	RMSEA [90% CI]	SRMR	Interpretation
M1	Second‐order CFA (formative‐composite)	1149.30	366	0.945	0.939	0.072 [0.068, 0.077]	0.043	Comparison model; discriminant validity failed (HTMT = 1.06)
M2	Bifactor CFA	699.11	348	0.958	0.951	0.050 [0.045–0.054]	0.027	Primary model; best fit

*Note:* The second‐order model was estimated in AMOS; the bifactor model was estimated in R (lavaan) with robust ML (MLR). The bifactor model includes one general factor and six orthogonal specific factors for the 26 reflective items. The structural dimension (formative) was excluded.

Abbreviation: CFA, confirmatory factor analysis.

#### 3.2.4. Confirmatory Factor Analysis

An initial second‐order CFA with formative structural composites demonstrated acceptable but suboptimal fit (χ^2^(366) = 1149.30, CFI = 0.945, TLI = 0.939, RMSEA = 0.072 [90% CI 0.068–0.077]). However, this model exhibited intractable discriminant validity failures (second‐order HTMT = 1.06; first‐order HTMT > 0.90 for several pairs; Supporting File 2, Table S3), indicating that the process and outcome dimensions were not empirically distinct [67].

Consequently, a bifactor CFA was estimated as the primary model (Figure 1). The bifactor model demonstrated acceptable fit: χ^2^(348) = 699.11, CFI = 0.958, TLI = 0.951, RMSEA = 0.050 (90% CI 0.045–0.054), SRMR = 0.027 (Table 3). All standardised factor loadings on the general factor were significant under MLR robust standard errors (*z* = 15.44–19.40, *p* < 0.001). General factor loadings ranged from 0.70 to 0.89 (*M* = 0.83), indicating strong saturation of the reflective items by the general factor. Specific factor loadings were generally lower (*λ* = 0.13–0.61), consistent with dominant unidimensionality (Supporting File 2, Table S4).

For the formative structural dimension, all three composites contributed significantly to the general factor (standardised weights: 0.251 [human resources], 0.208 [facilities and equipment], 0.454 [management systems]; all *p* ≤ 0.001). At the composite level, variance inflation factors among the three formative structural composites ranged from 1.62 to 2.10. The structural dimension significantly predicted the general factor (*β* = 0.78, *p* < 0.001), consistent with Donabedian’s causal chain [22].

Province‐level ICC(1) for the total score was 0.009; city‐level ICC(1) was 0.004. In conservative scenario analyses (ICC = 0.05–0.15), adjusted fit remained acceptable and all loadings remained significant (minimum adjusted critical ratio [CR] = 10.08; Supporting File 2, Table S5) [75, 76]. The MLR‐scaled χ^2^ test yielded *p* < 0.001. Because this procedure tests the exact‐fit hypothesis (i.e., Σ = Σ(θ)), significant *p* values are common in large samples (*N* = 410) where even trivial model misspecification becomes statistically detectable [70]. Consistent with current recommendations, model fit was evaluated primarily through approximate fit indices (CFI, TLI, RMSEA, SRMR) rather than the exact‐fit χ^2^ test alone [58, 70].

#### 3.2.5. Construct Validity and the Bifactor Model

The bifactor model demonstrated acceptable fit (Table 3). Construct validity indices were as follows (Table 4): (1) The ECV for the general factor was 0.869, exceeding the 0.70 threshold. This supports the scale’s essential unidimensionality and explains why traditional discriminant validity metrics (HTMT) failed—the subdimensions share a dominant common core rather than representing distinct constructs. (2) The hierarchical omega for the general factor (ωH = 0.963) exceeded the 0.80 threshold, supporting interpretation of the 26‐item reflective total score as the primary metric. (3) The hierarchical omega for the specific factors (ωHS range 0.05–0.19) was substantially below the 0.50 threshold. This indicates that the six subdimensions are not empirically distinct reflective constructs. They were retained as descriptive item groupings to preserve the Delphi‐derived conceptual taxonomy, and their independent statistical reliability is insufficient for standalone interpretation or for identifying unit‐level care gaps.

**TABLE 4:** Bifactor model structure and reliability indices for the NMC‐PSD Scale (*N* = 410).

Factor type	Factor name	No. of items	ECV	ω/ωH	ωHS	Interpretation
General	NMC‐PSD General Capacity	26	0.869	0.963	—	Supports unweighted total score as primary metric
Specific	Assessment	4	—	—	0.19^a^	Not independently reliable; descriptive grouping only
Specific	Practice	7	—	—	0.13^a^	Not independently reliable; descriptive grouping only
Specific	Education	4	—	—	0.05^a^	Not independently reliable; descriptive grouping only
Specific	Follow‐up	3	—	—	0.08^a^	Not independently reliable; descriptive grouping only
Specific	Nurse outcomes	3	—	—	0.11^a^	Not independently reliable; descriptive grouping only
Specific	Patient outcomes	5	—	—	0.12^a^	Not independently reliable; descriptive grouping only
Formative composite	Structure	9	—	—	—	Formative specification; validity rests on content validity and expert consensus

*Note:* Thresholds: ECV ≥ 0.70 indicates essential unidimensionality; ωH ≥ 0.80 indicates reliable total score; ωHS ≥ 0.50 indicates reliable subscale interpretation [43]. The ωH and ECV values apply strictly to the 26 reflective process and outcome items; the nine formative structural items were excluded from the reflective bifactor validation. The structural dimension was specified as formative and excluded from the reflective bifactor validation.

Abbreviations: ωH, omega hierarchical; ωHS, omega hierarchical subscale; ECV, explained common variance.

^a^Exact values were retrieved from the bifactorIndices() function in the BifactorIndicesCalculator package.

These results support the validity of the 26‐item reflective core of the NMC‐PSD Scale as a primarily unidimensional instrument. Within the reflective domain, the 26‐item reflective total score is the only psychometrically robust metric; subscale profiles offer conceptual or descriptive grouping value only and must not be used for subscale‐level inference. The nine formative structural indicators are reported separately as descriptive resource inputs. Their retention rests on content validity (I‐CVI ≥ 0.80; S‐CVI/Ave = 0.93). The test–retest ICCs of 0.98 (total scale) and 0.99 (human resources composite) indicate stability but do not constitute independent reliability evidence for all nine formative indicators as a complete component.

#### 3.2.6. Reliability

Subdimension‐level reliability coefficients were 0.97 for the process dimension and 0.95 for the outcome dimension. Cronbach’s *α* is not reported for the structural dimension or its subdimensions given their formative specification [42]. Test–retest reliability over a 2‐week interval was adequate, with an ICC of 0.98 (95% CI 0.96–0.99) for the total scale; the formative human resources composite also showed high stability (ICC = 0.99, 95% CI 0.98–0.99, *n* = 41).

### 3.3. Descriptive Analysis of Scale Scores

Item scores and descriptive statistics are shown in Table 5. For the primary validated metric (26‐item reflective total, range 26–130), the mean reflective total score was 54.71 (SD = 18.40). The 35‐item unweighted total (range 35–175), which includes nine formative structural indicators for descriptive completeness, had a mean of 73.68 (SD = 22.46). When converted to the exploratory weighted index (full score = 500) for descriptive reference, the mean was 207.87 (SD = 66.41). Unweighted item means ranged from 1.73 to 3.21; corresponding exploratory weighted derivatives are presented in Table 5 for completeness.

**TABLE 5:** Descriptive analysis of items in the NMC‐PSD scale (*N* = 410).

Items	Mean ± SD	Item weight^∗^	Weighted mean ± SD
Dimension 1	20.95 ± 5.78		73.01 ± 22.12
1.1	12.70 ± 3.46		27.95 ± 8.30
1.1.1	2.31 ± 1.03	2.24	5.02 ± 2.49
1.1.2	3.21 ± 0.85	2.24	7.24 ± 2.01
1.1.3	2.03 ± 0.77	2.24	4.32 ± 1.90
1.1.4	2.30 ± 0.88	2.24	5.02 ± 2.19
1.1.5	2.85 ± 1.08	2.24	6.35 ± 2.58
1.2	4.30 ± 1.61		22.75 ± 8.64
1.2.1	2.56 ± 1.02	5.32	13.65 ± 5.35
1.2.2	1.73 ± 0.95	5.32	9.09 ± 5.20
1.3	3.95 ± 1.62		22.31 ± 8.84
1.3.1	1.97 ± 0.87	5.60	11.16 ± 4.78
1.3.2	1.97 ± 0.86	5.60	11.16 ± 4.73
Dimension 2	36.81 ± 12.48		69.60 ± 24.57
2.1	8.48 ± 2.85		17.08 ± 5.99
2.1.1	2.02 ± 0.82	2.13	4.07 ± 1.71
2.1.2	2.03 ± 0.81	2.13	4.09 ± 1.71
2.1.3	2.23 ± 0.88	2.13	4.51 ± 1.87
2.1.4	2.19 ± 0.76	2.13	4.41 ± 1.60
2.2	13.78 ± 4.98		17.81 ± 7.19
2.2.1	1.94 ± 0.78	1.28	2.60 ± 1.21
2.2.2	1.92 ± 0.74	1.28	2.60 ± 1.16
2.2.3	1.98 ± 0.79	1.28	2.67 ± 1.20
2.2.4	1.95 ± 0.77	1.28	2.64 ± 1.19
2.2.5	1.98 ± 0.82	1.28	2.66 ± 1.24
2.2.6	1.94 ± 0.78	1.28	2.61 ± 1.20
2.2.7	2.07 ± 0.86	0.85	2.03 ± 0.78
2.3	8.23 ± 3.03		16.58 ± 6.27
2.3.1	1.98 ± 0.82	2.13	3.97 ± 1.70
2.3.2	2.19 ± 0.82	2.13	4.42 ± 1.74
2.3.3	2.03 ± 0.80	2.13	4.07 ± 1.63
2.3.4	2.03 ± 0.84	2.13	4.11 ± 1.78
2.4	6.23 ± 2.49		18.12 ± 7.07
2.4.1	2.03 ± 0.87	2.91	6.08 ± 2.62
2.4.2	2.08 ± 0.90	2.91	6.23 ± 2.69
2.4.3	2.22 ± 0.90	2.49	5.81 ± 2.26
Dimension 3	15.92 ± 5.67		65.26 ± 24.57
3.1	6.08 ± 2.33		34.44 ± 13.22
3.1.1	2.06 ± 0.81	5.93	12.38 ± 4.83
3.1.2	2.00 ± 0.84	5.93	11.97 ± 5.01
3.1.3	2.02 ± 0.82	5.08	10.10 ± 4.08
3.2	9.85 ± 3.51		30.82 ± 12.13
3.2.1	1.85 ± 0.77	3.22	5.74 ± 2.63
3.2.2	2.01 ± 0.77	3.22	6.30 ± 2.67
3.2.3	2.03 ± 0.78	3.22	6.38 ± 2.72
3.2.4	1.95 ± 0.78	3.22	6.09 ± 2.70
3.2.5	2.01 ± 0.78	3.22	6.31 ± 2.72
Reflective total (26 items)	**54.71 ± 18.40**		
Total	**73.68 ± 22.46**		**207.87 ± 66.41**

*Note:* Bold values indicate the reflective total for 26 items and the total score of the scale.

Abbreviation: SD, standard deviation.

^∗^The item weights formed after optimising the original evaluation indicators after expert consensus. For comparability with the original inventory audit, item scores are still described as mean ± SD. The unweighted mean scores (Column 2) serve as the primary psychometrically validated scale metrics. The weighted scores (Column 4) are exploratory derivatives intended solely for descriptive comparison with prior checklist‐based audits and do not carry validated psychometric properties from this study.

Weighted item scores, calculated according to the original checklist weights, ranged from 2.03 to 13.65. The highest weighted score was observed for item 1.2.1, ‘Essential dysphagia care and rehabilitation equipment available and functional’ (13.65, SD 5.35). The lowest weighted score was for item 2.2.7, ‘Personalised feeding plan effectively implemented’ (2.03, SD 0.78). Under the unweighted condition, the highest‐scoring item was item 1.1.2, ‘Physician‐to‐bed ratio meets patient needs’ (mean 3.21, SD 0.85), whereas the lowest‐scoring was item 1.2.2, ‘Complete and functional emergency equipment’ (mean 1.73, SD 0.95). All subsequent item‐level analyses used unweighted Likert means [52].

## 4. Discussion

This study developed and preliminarily evaluated the NMC‐PSD Scale in a multiprovince convenience sample of 410 nurses in China. The bifactor model was specified post hoc after the a priori second‐order CFA showed discriminant validity failures. As both model respecification and estimation used the same sample, the structural findings require independent cross‐validation. The 26‐item reflective core demonstrated acceptable psychometric properties, including adequate content validity, acceptable bifactor model fit (χ^2^(348) = 699.11, CFI = 0.958, TLI = 0.951, RMSEA = 0.050, SRMR = 0.027), dominant general‐factor unidimensionality (ECV = 0.869, ωH = 0.963) and adequate internal consistency and test–retest reliability. The nine formative structural indicators were validated separately through content validity and expert consensus. Traditional convergent and discriminant validity indices (AVE/CR, HTMT) are retained as supporting information, as the bifactor framework supersedes them under the present specification. These findings suggest that the NMC‐PSD Scale is a psychometrically acceptable instrument for assessing nursing management capacity in dysphagia care.

The NMC‐PSD Scale originated from an operational checklist that was adapted to a self‐report format. The content validity indices (S‐CVI/Ave = 0.93; I‐CVI = 0.80–1.00) reflect adequate expert agreement on item relevance. During pilot testing, the scale was completed in a median of 3.39 min (IQR 2.16–4.41), indicating acceptable clinical feasibility. Two items were refined to enhance operational specificity: item 2.2.1 was elaborated with exemplar feeding routes, and item 3.2.1 was anchored to electronic health record documentation. These iterative modifications underscore the importance of explicit operational definitions when translating checklist indicators into self‐report items.

The structure–process–outcome model [22] identifies nursing management deficiencies (e.g. inadequate resource allocation). The NMC‐PSD Scale follows a mixed measurement model consistent with Donabedian’s framework [22]. The structural dimension is specified as a formative second‐order construct: its components (human resources, facilities and management systems) are heterogeneous resource inputs that jointly constitute management capacity rather than interchangeable reflections of a single latent resource factor [33, 42]. Under formative specification, causal indicators are not required to correlate with one another or to share a common latent source; rather, each indicator represents a distinct, nonsubstitutable input to the construct [34]. Applying conventional reflective‐scale metrics (e.g. CITC, standardised factor loading) to formative indicators risks construct underrepresentation by deleting content‐necessary but statistically weak items [65]. Consequently, when adapting Donabedian‐based checklists into self‐report scales, traditional reflective psychometric criteria must not be mechanically applied to formative structural indicators.

The bifactor respecification resolves the discriminant validity paradox observed in the initial second‐order CFA (HTMT = 1.06) by attributing the apparent correlation between process and outcome to a dominant general factor rather than to a direct causal path. Donabedian’s S‐P‐O model thus functions as a conceptual taxonomy for item generation and clinical screening, while the statistical model aligns with the empirical structure of nurse perceptions—namely, a unified global evaluation of general capacity adequacy [22, 36]. As detailed in the Results, the six specific factors lack sufficient independent reliability (ωHS < 0.50) for standalone interpretation [36]; they are retained solely as descriptive item groupings reflecting the Delphi‐derived taxonomy. Given their very low independent reliability (ωHS = 0.05–0.19), these groupings must not be used to identify unit‐level care gaps. Researchers and administrators should therefore report the 26‐item reflective total score as the primary outcome, presenting subscale profiles only as descriptive content groupings without diagnostic interpretation.

The retention of item 1.1.2 (physician‐to‐bed ratio) illustrates the divergence between formative and reflective logic. Its low corrected item‐total correlation is deliberate. The item captures physician staffing adequacy, a resource input that is conceptually distinct from nurse staffing, equipment and management systems. Under formative logic, such heterogeneity is expected and desirable [33, 42]. Its removal would risk construct underrepresentation, and its perfect content validity (I‐CVI = 1.00) together with the lowest variance inflation factor (1.32) within the human resources composite confirms its unique, nonredundant contribution. This example underscores why traditional reflective psychometric criteria must not be mechanically applied to formative structural indicators when adapting Donabedian‐based checklists.

Internal consistency was evaluated separately for the reflective dimensions. The process dimension (18 items) showed *α* = 0.97 and the outcome dimension (8 items) showed *α* = 0.95 [77]. As the nine formative indicators are not expected to covary, a total‐scale *α* was not computed for the 35‐item composite [34]. However, the elevated *α* for the process dimension partly reflects scale length rather than unidimensionality alone [78, 79]. Their aggregation under a single process dimension is justified by the Donabedian framework rather than by a unidimensional measurement model.

To maintain interpretational clarity, our primary item‐level analysis used unweighted Likert means, which represent the psychometrically validated metric. The exploratory weighted derivatives are referenced descriptively to bridge to the original checklist framework. Regarding the scoring approach, the item with the highest weighted score (1.2.1) did not emerge as the top‐performing item under unweighted scoring, and the item with the lowest weighted score (2.2.7) differed from the lowest unweighted item (1.2.2). This discrepancy indicates that weighted rankings partly reflect the original scoring design rather than actual performance. To avoid this confounding, item‐level interpretation in this study was based on unweighted Likert means, whereas weighted scores were reserved solely for calculation of the overall management capacity index [52, 80]. This dual approach preserves the original checklist’s evidence‐based weighting at the composite level while ensuring interpretability at the item level as an exploratory descriptive index.

Physician‐to‐bed ratio scored relatively high. This finding is consistent with prior research suggesting that Chinese public hospitals may emphasise staffing metrics that are readily auditable under accreditation schemes [81, 82]. Because our scale measures nurses’ subjective perceptions, this high score reflects perceived adequacy of physician staffing, not objective compliance. Adequate physician staffing is necessary but not sufficient for good nursing management [83, 84]; thus, a comparatively favourable score should not be interpreted as evidence of superior nursing practice or patient outcomes [82].

Emergency equipment functionality scored lower. This pattern is consistent with documented problems in medical device maintenance, including fragmented accountability across departments [85], the possibility that inspections may prioritise verifying physical presence and external appearance over comprehensive functional performance testing [86], and delayed equipment replacement cycles [87]. From frontline nurses’ perspective, these low scores suggest that equipment maintenance is seen as a system‐level vulnerability rather than a ward‐specific issue. Although self‐report data cannot pinpoint the exact governance mechanisms behind these perceptions, the observed pattern raises the hypothesis that safety audits may need to test actual equipment function rather than rely solely on inventory checks [88], although such interpretations warrant objective verification in future research.

## 5. Limitations and Future Perspectives

This study has several limitations. First, the self‐report design introduces potential response bias; structural items reflect nurses’ perceptions rather than objective administrative indicators. Future research should validate these against institutional records. Therefore, interpretations linking scores to specific institutional causes should be treated as informed by the broader literature rather than as directly proven by these perceptual data.

Second, the sample was predominantly female, recruited primarily from tertiary hospitals and geographically concentrated in southwestern China, which restricts generalisability; subgroup sizes precluded multigroup CFA and measurement invariance testing. As a unit‐level self‐assessment completed by individual nurses, scores inherently reflect the respondent’s vantage point within the organisational hierarchy. Future research should examine inter‐rater agreement among nurses of varying ranks within the same unit and establish the minimum number of respondents required for stable unit‐level estimates. Cross‐validation in secondary hospital settings, where structural resource disparities are more pronounced, would further clarify the performance of structural indicators across diverse contexts [89, 90].

Third, although we employed maximum likelihood estimation with Bollen–Stine bootstrap to handle non‐normality, the ordinal nature of the five‐point Likert data suggests that categorical estimators such as weighted least squares mean and variance adjusted may provide more accurate parameter estimates and should be employed in future cross‐validation studies to confirm the stability of the present findings.

Fourth, both the structural dimension (specified as formative) and the six specific factors (with low ωHS = 0.05–0.19) carry interpretation caveats. As noted in the Results, the ωH estimate applies only to the 26‐item reflective core; the nine formative structural items rely on content validity rather than internal consistency metrics. Future validation should examine whether it can be integrated into a comprehensive bifactor specification through formative–bifactor hybrid models, and structural indicators should be validated against objective administrative records (e.g. actual staffing ratios, equipment audit logs) to establish criterion validity [42, 65]. The low ωHS values limit the interpretive utility of subscale profiles. Although the six subdimensions are retained to preserve the Delphi‐derived conceptual taxonomy, their independent statistical reliability is below the threshold for standalone reflective interpretation. Researchers requiring subscale‐level inference should consider longer subscale‐specific item pools in future scale extensions.

Fifth, the cross‐sectional design precluded testing predictive validity against clinical outcomes (e.g. pneumonia rates, length of stay). Future longitudinal research should examine whether process and outcome dimensions differentially predict these endpoints, while interpreting such predictive distinctions alongside the dominant general factor given the low ωHS values observed.

Finally, although we provided interpretive guidance for the weighted composite score, future research should establish criterion‐referenced cut‐offs for clinical benchmarking. Criterion‐related validation studies linking the exploratory weighted index to objective administrative metrics (e.g. pneumonia rates, screening adherence logs) are warranted before its use as a formal audit benchmark.

## 6. Implications for Nursing Management

The 26‐item reflective total score is the psychometrically validated primary metric. It supports individual‐level inference: quantifying a nurse’s perception of general management capacity in his or her unit. The scale can be used for needs assessment and evaluating pre–post intervention changes. The nine structural indicators should be reported separately as descriptive resource profiles. The six subscale groupings are retained as conceptual labels only; given their low reliability (ωHS = 0.05–0.19), they must not be used for subscale‐level inference or identifying unit‐level care gaps.

Several constraints currently preclude organisational‐level application. The scale was validated as an individual‐level measure; aggregation to unit‐level scores requires evidence of within‐unit agreement and aggregation reliability, which were unavailable because hospital identifiers were not collected. The bifactor respecification was developed in the same sample; independent cross‐validation is necessary.

Before the scale can support organisational benchmarking, the following are warranted: multilevel measurement invariance testing; within‐unit agreement thresholds; criterion‐related validation against objective indicators (e.g. aspiration pneumonia rates); and cross‐validation in diverse hospital settings. Until then, organisational‐level benchmarking claims should be avoided.

## 7. Conclusions

The NMC‐PSD Scale was developed and preliminarily evaluated, demonstrating acceptable reliability and validity for individual‐level assessment. We recommend the 26‐item reflective total score (range 26–130) as the primary validated metric, supported by bifactor reliability (ωH = 0.963, ECV = 0.869). Nine formative structural indicators are reported separately as descriptive resource profiles. As the bifactor respecification was developed in the same sample, independent cross‐validation is warranted. This standardised tool supports care consistency in routine dysphagia management. The exploratory weighted index, derived from the original Delphi‐AHP framework, serves as a descriptive bridge to prior checklist‐based audits but requires future criterion‐related validation against objective administrative indicators (e.g. aspiration pneumonia rates, screening adherence logs) before its use as a formal audit benchmark. Further testing of responsiveness, inter‐rater reliability and cross‐setting measurement invariance is warranted to support broader implementation in training evaluation and quality improvement initiatives.

## Author Contributions

Concept and design: Ying Zou, Yiwen Chen, Changyue Gao and Changyan Yuan. Acquisition, access and analysis of data: Ying Zou, Hongyu Chen, Yiwen Chen and Lingyi Liao. Drafting of the manuscript: Ying Zou and Yiwen Chen. Critical revision of the manuscript for important intellectual content: Lingyi Liao, Changyue Gao and Changyan Yuan. Administrative, technical, or material support: Changyue Gao and Changyan Yuan. All authors contributed to the study conceptualisation, and critically reviewed and revised the manuscript draft.

## Funding

This study was supported by the Chongqing Science and Health Joint Youth Project (Grant No. 2024QNXM002).

## Disclosure

The funder had no role in study design, data collection, analysis or interpretation; the writing of the manuscript; or the decision to submit for publication. All authors approved the final version for submission.

## Conflicts of Interest

The authors declare no conflicts of interest.

## Supporting Information

Additional supporting information can be found online in the Supporting Information section.

## Supporting information


**Supporting Information** Supporting File 1: NMC‐PSD Scale and Scoring System. Supporting File 2: Supporting Statistical Analyses (Model Comparison, Bifactor Loadings and Robustness Checks). Supporting File 3: Two‐Round Delphi Consultation: Procedure, Round‐Specific Changes and Content Validity Indices. Supporting File 4: Reproducible lavaan Model Syntax and Output for the Bifactor Analysis. Supporting File 4_STROBE Checklist.

## Data Availability

The data that support the findings of this study are available from the corresponding author upon reasonable request.
