# Supplementary material for: Development and Psychometric Evaluation of the Nursing Management Capacity Scale for Poststroke Dysphagia Based on the Donabedian Model
Source: J Nurs Manag. 2026 Sep 20;2026:9248618. doi: 10.1155/jonm/9248618 (PMC13591012; doi:10.1155/jonm/9248618)
Supplement: Supplementary file 1 [file JONM-2026-9248618-s001.zip › Supplementary File 1_ NMC-PSD Scale and Scoring System.docx]

**Supplementary File 1: NMC-PSD Scale and Scoring System**

**Table S1. Hierarchical Structure and Weight Distribution of the NMC-PSD Scale’s Indicators**

| **Primary items**  **(Weight)** | **Secondary items**  **(Weight)** | **Tertiary items** | **Weight of Tertiary Items** | **Overall Weight（%）** | $\boldsymbol{P}_{\boldsymbol{i}}$***** |
| --- | --- | --- | --- | --- | --- |
| Structural dimensions (33.051%) | Human resource allocation  (33.898%) | Nurse-to-bed ratio meets patient needs | 20% | 22407.26 | 2.24 |
|  |  | Physician-to-bed ratio meets patient needs | 20% | 22407.26 | 2.24 |
|  |  | Appropriate allocation of nursing staff by level | 20% | 22407.26 | 2.24 |
|  |  | High-functioning MDT^1^ | 20% | 22407.26 | 2.24 |
|  |  | Therapist-to-bed ratio meets patient needs | 20% | 22407.26 | 2.24 |
|  | Facilities and equipment  (32.203%) | Essential dysphagia care/rehabilitation equipment available & functional | 50% | 53217.07 | 5.32 |
|  |  | Complete & functional emergency equipment | 50% | 53217.07 | 5.32 |
|  | Management systems  (33.898%) | Clear dysphagia screening/assessment protocol | 50% | 56018.14 | 5.60 |
|  |  | Practical & actionable aspiration emergency plan | 50% | 56018.14 | 5.60 |
| Process dimensions (33.898%) | Implementation rates of nursing assessment  (25.157%) | Correct screening tool use for new stroke patients | 25% | 21319.30 | 2.13 |
|  |  | MDT^1^ correctly co-assesses dysphagia | 25% | 21319.30 | 2.13 |
|  |  | Correct nutritional risk screening tool use | 25% | 21319.30 | 2.13 |
|  |  | Correct oral hygiene assessment & care solution selection | 25% | 21319.30 | 2.13 |
|  | Nursing practices  (25.157%) | Correct feeding route selection | 15% | 12791.58 | 1.28 |
|  |  | Correct feeding posture selection | 15% | 12791.58 | 1.28 |
|  |  | Correct judgment of feeding pace | 15% | 12791.58 | 1.28 |
|  |  | Correct guidance on food consistency | 15% | 12791.58 | 1.28 |
|  |  | Correct judgment of feeding utensils | 15% | 12791.58 | 1.28 |
|  |  | Correct oral care procedure | 15% | 12791.58 | 1.28 |
|  |  | Personalized feeding plan effectively implemented | 10% | 8527.72 | 0.85 |
|  | Health education  (25.157%) | Comprehensive education for inpatients with dysphagia | 25% | 21319.30 | 2.13 |
|  |  | Dysphagia knowledge education achieves expected outcomes | 25% | 21319.30 | 2.13 |
|  |  | Patients master rehabilitation techniques post-education | 25% | 21319.30 | 2.13 |
|  |  | Detailed discharge rehabilitation guidance provided | 25% | 21319.30 | 2.13 |
|  | Follow-up management  (24.528%) | Timely patient follow-up completion | 35% | 29100.76 | 2.91 |
|  |  | Accurate follow-up documentation | 35% | 29100.76 | 2.91 |
|  |  | High patient follow-up appointment adherence | 30% | 24943.50 | 2.49 |
| Outcome dimensions (33.051%) | Nurse-related outcomes  (51.282%) | Strong nurse dysphagia knowledge | 35% | 59322.25 | 5.93 |
|  |  | Proficient nurse dysphagia skills | 35% | 59322.25 | 5.93 |
|  |  | High physician satisfaction with nursing care | 30% | 50847.64 | 5.08 |
|  | Patient-related outcomes  (48.718%) | Near-absence of dysphagia-related adverse events | 20% | 32203.57 | 3.22 |
|  |  | High patient/family satisfaction with rehabilitation care | 20% | 32203.57 | 3.22 |
|  |  | High patient compliance with rehabilitation therapy | 20% | 32203.57 | 3.22 |
|  |  | Near-absence of aspiration pneumonia | 20% | 32203.57 | 3.22 |
|  |  | Significant improvement in swallowing function | 20% | 32203.57 | 3.22 |

*Note:* MDT, multidisciplinary team. *$P_{i}$, item weight percentage, was obtained by normalizing the product of the three-level Delphi weights. Scores for the five response options were derived from a maximum base of $P_{i}$×5 using proportional coefficients of 1.0 : 0.8 : 0.6 : 0.4 : 0.2 and rounded to the nearest integer. Scores for all items are in Table S2.

**Table S2a: Nursing Management Capacity in Post-Stroke Dysphagia (NMC-PSD) Scale***

| **Item** | **Options** | | | | |  |
| --- | --- | --- | --- | --- | --- | --- |
| **1. Structural Factors** | **Strongly Agree** | **Agree** | **Neutral** | **Disagree** | **Strongly Disagree** |  |
| **1.1 Human Resource Allocation** | | | | | | |
| 1.1.1 Nurse-to-bed ratio meets patient needs |  |  |  |  |  |  |
| 1.1.2 Physician-to-bed ratio meets patient needs |  |  |  |  |  |  |
| 1.1.3 Appropriate allocation of nursing staff by level |  |  |  |  |  |  |
| 1.1.4 High-functioning MDT |  |  |  |  |  |  |
| 1.1.5 Therapist-to-bed ratio meets patient needs |  |  |  |  |  |  |
| **1.2 Facilities & Equipment** | | | | | | |
| 1.2.1 Essential dysphagia care/rehabilitation equipment available & functional |  |  |  |  |  |  |
| 1.2.2 Complete & functional emergency equipment |  |  |  |  |  |  |
| **1.3 Management Systems** | | | | | | |
| 1.3.1 Clear dysphagia screening/assessment protocol |  |  |  |  |  |  |
| 1.3.2 Practical & actionable aspiration emergency plan |  |  |  |  |  |  |
| **2. Process Indicators** | | | | | | |
| **2.1 Nursing Assessment** | | | | | | |
| 2.1.1 Correct screening tool use for new stroke patients |  |  |  |  |  |  |
| 2.1.2 MDT correctly co-assesses dysphagia |  |  |  |  |  |  |
| 2.1.3 Correct nutritional risk screening tool use |  |  |  |  |  |  |
| 2.1.4 Correct oral hygiene assessment & care solution selection |  |  |  |  |  |  |
| **2.2 Nursing Practice** | | | | | | |
| 2.2.1 Correct feeding route selection (oral, IOE, NGT, etc.) |  |  |  |  |  |  |
| 2.2.2 Correct feeding posture selection |  |  |  |  |  |  |
| 2.2.3 Correct judgment of feeding pace |  |  |  |  |  |  |
| 2.2.4 Correct guidance on food consistency |  |  |  |  |  |  |
| 2.2.5 Correct judgment of feeding utensils |  |  |  |  |  |  |
| 2.2.6 Correct oral care procedure |  |  |  |  |  |  |
| 2.2.7 Personalized feeding plan effectively implemented^#^ |  |  |  |  |  |  |
| **2.3 Patient Education** | | | | | | |
| 2.3.1 Comprehensive education for inpatients with dysphagia |  |  |  |  |  |  |
| 2.3.2 Dysphagia knowledge education achieves expected outcomes |  |  |  |  |  |  |
| 2.3.3 Patients master rehabilitation techniques post-education |  |  |  |  |  |  |
| 2.3.4 Detailed discharge rehabilitation guidance provided |  |  |  |  |  |  |
| **2.4 Follow-up Management** | | | | | | |
| 2.4.1 Timely patient follow-up completion |  |  |  |  |  |  |
| 2.4.2 Accurate follow-up documentation |  |  |  |  |  |  |
| 2.4.3 High patient follow-up appointment adherence |  |  |  |  |  |  |
| **3. Outcome Indicators** | | | | | | |
| **3.1 Nurse Outcomes** | | | | | | |
| 3.1.1 Strong nurse dysphagia knowledge |  |  |  |  |  |  |
| 3.1.2 Proficient nurse dysphagia skills |  |  |  |  |  |  |
| 3.1.3 High physician satisfaction with nursing care |  |  |  |  |  |  |
| **3.2 Patient Outcomes** | | | | | | |
| 3.2.1 Near-absence of dysphagia-related adverse events (per EMR) |  |  |  |  |  |  |
| 3.2.2 High patient/family satisfaction with rehabilitation care |  |  |  |  |  |  |
| 3.2.3 High patient compliance with rehabilitation therapy |  |  |  |  |  |  |
| 3.2.4 Near-absence of aspiration pneumonia |  |  |  |  |  |  |
| 3.2.5 Significant improvement in swallowing function |  |  |  |  |  |  |

*Note:* MDT, multidisciplinary team; IOE, oro-esophageal tube feeding; NGT, nasogastric tube; EMR, electronic medical record.

^*^If unsure of exact numbers, provide your best estimate based on typical shifts.

^#^ Since items in this category have relatively low weights, the “neutral” and “disagree” response options yield identical integer scores upon rounding.

**Table S2b: Nursing Management Capacity in Post-Stroke Dysphagia (NMC-PSD) Scale** **(Weighted)**

| **Item** | **Options** | | | | | **Score** |
| --- | --- | --- | --- | --- | --- | --- |
| **1. Structural Factors** | **Strongly Agree** | **Agree** | **Neutral** | **Disagree** | **Strongly Disagree** |  |
| **1.1 Human Resource Allocation** | | | | | | |
| 1.1.1 Nurse-to-bed ratio meets patient needs | 11 | 9 | 7 | 4 | 2 |  |
| 1.1.2 Physician-to-bed ratio meets patient needs | 11 | 9 | 7 | 4 | 2 |  |
| 1.1.3 Appropriate allocation of nursing staff by level | 11 | 9 | 7 | 4 | 2 |  |
| 1.1.4 High-functioning MDT | 11 | 9 | 7 | 4 | 2 |  |
| 1.1.5 Therapist-to-bed ratio meets patient needs | 11 | 9 | 7 | 4 | 2 |  |
| **1.2 Facilities & Equipment** | | | | | | |
| 1.2.1 Essential dysphagia care/rehabilitation equipment available & functional | 27 | 22 | 16 | 11 | 5 |  |
| 1.2.2 Complete & functional emergency equipment | 27 | 22 | 16 | 11 | 5 |  |
| **1.3 Management Systems** | | | | | | |
| 1.3.1 Clear dysphagia screening/assessment protocol | 28 | 22 | 17 | 11 | 6 |  |
| 1.3.2 Practical & actionable aspiration emergency plan | 28 | 22 | 17 | 11 | 6 |  |
| **2. Process Indicators** | | | | | | |
| **2.1 Nursing Assessment** | | | | | | |
| 2.1.1 Correct screening tool use for new stroke patients | 11 | 9 | 7 | 4 | 2 |  |
| 2.1.2 MDT correctly co-assesses dysphagia | 11 | 9 | 7 | 4 | 2 |  |
| 2.1.3 Correct nutritional risk screening tool use | 11 | 9 | 7 | 4 | 2 |  |
| 2.1.4 Correct oral hygiene assessment & care solution selection | 11 | 9 | 7 | 4 | 2 |  |
| **2.2 Nursing Practice** | | | | | | |
| 2.2.1 Correct feeding route selection (oral, IOE, NGT, etc.) | 6 | 5 | 4 | 2 | 1 |  |
| 2.2.2 Correct feeding posture selection | 6 | 5 | 4 | 2 | 1 |  |
| 2.2.3 Correct judgment of feeding pace | 6 | 5 | 4 | 2 | 1 |  |
| 2.2.4 Correct guidance on food consistency | 6 | 5 | 4 | 2 | 1 |  |
| 2.2.5 Correct judgment of feeding utensils | 6 | 5 | 4 | 2 | 1 |  |
| 2.2.6 Correct oral care procedure | 6 | 5 | 4 | 2 | 1 |  |
| 2.2.7 Personalized feeding plan effectively implemented^#^ | 4 | 3 | 2 | 2 | 1 |  |
| **2.3 Patient Education** | | | | | | |
| 2.3.1 Comprehensive education for inpatients with dysphagia | 11 | 9 | 7 | 4 | 2 |  |
| 2.3.2 Dysphagia knowledge education achieves expected outcomes | 11 | 9 | 7 | 4 | 2 |  |
| 2.3.3 Patients master rehabilitation techniques post-education | 11 | 9 | 7 | 4 | 2 |  |
| 2.3.4 Detailed discharge rehabilitation guidance provided | 11 | 9 | 7 | 4 | 2 |  |
| **2.4 Follow-up Management** | | | | | | |
| 2.4.1 Timely patient follow-up completion | 15 | 12 | 9 | 6 | 3 |  |
| 2.4.2 Accurate follow-up documentation | 15 | 12 | 9 | 6 | 3 |  |
| 2.4.3 High patient follow-up appointment adherence | 12 | 10 | 7 | 5 | 2 |  |
| **3. Outcome Indicators** | | | | | | |
| **3.1 Nurse Outcomes** | | | | | | |
| 3.1.1 Strong nurse dysphagia knowledge | 30 | 24 | 18 | 12 | 6 |  |
| 3.1.2 Proficient nurse dysphagia skills | 30 | 24 | 18 | 12 | 6 |  |
| 3.1.3 High physician satisfaction with nursing care | 25 | 20 | 15 | 10 | 5 |  |
| **3.2 Patient Outcomes** | | | | | | |
| 3.2.1 Near-absence of dysphagia-related adverse events (per EMR) | 16 | 13 | 10 | 6 | 3 |  |
| 3.2.2 High patient/family satisfaction with rehabilitation care | 16 | 13 | 10 | 6 | 3 |  |
| 3.2.3 High patient compliance with rehabilitation therapy | 16 | 13 | 10 | 6 | 3 |  |
| 3.2.4 Near-absence of aspiration pneumonia | 16 | 13 | 10 | 6 | 3 |  |
| 3.2.5 Significant improvement in swallowing function | 16 | 13 | 10 | 6 | 3 |  |
| **Total**^†^ | | | | | |  |

*Note:* MDT, multidisciplinary team; IOE, oro-esophageal tube feeding; NGT, nasogastric tube; EMR, electronic medical record. The 26-item unweighted reflective total score (sum of the 26 reflective Process and Outcome items, range 26–130) serves as the primary psychometrically validated metric. The nine formative structural indicators are reported separately as descriptive resource inputs, and the weighted scores above are exploratory descriptive derivatives for comparison with prior checklist-based audits.

^*^If unsure of exact numbers, provide your best estimate based on typical shifts.

^#^ Since items in this category have relatively low weights, the “neutral” and “disagree” response options yield identical integer scores upon rounding.

^†^ The maximum possible score for each item varies, as items inherit distinct weights from the original scale. The table above can be used directly for scoring; the detailed weight calculation process can be found in Table S1. The scale adopts an unconventional scoring method, which demands greater care when calculating the total score. Thus, automated scoring is recommended to minimize human error.
