# Supplementary material for: Development and Psychometric Evaluation of the Nursing Management Capacity Scale for Poststroke Dysphagia Based on the Donabedian Model
Source: J Nurs Manag. 2026 Sep 20;2026:9248618. doi: 10.1155/jonm/9248618 (PMC13591012; doi:10.1155/jonm/9248618)
Supplement: Supplementary file 1 [file JONM-2026-9248618-s001.zip › Supplementary File 2_Supplementary Statistical Analyses(Model Comparison, Bifactor Loadings, and Robustness Checks).docx]

**Supplementary File 2**

**Table S3.** **Model Fit Indices for Sensitivity Analyses (*N* = 410)**

| **Model** | **χ²** | **df** | **CFI** | **TLI** | **RMSEA** | **Key Finding** |
| --- | --- | --- | --- | --- | --- | --- |
| A. Correlated first-order* | 2708.16 | 533 | 0.856 | 0.839 | 0.100 | Purely first-order structure cannot adequately represent the data, supporting the hierarchical specification |
| B. Formative-composite second-order | 1149.30 | 366 | 0.945 | 0.939 | 0.072 | Second-order HTMT = 1.06; first-order HTMT > 0.90 for several pairs, not satisfied. |
| C. Model B excluding Item 1.1.2 | 1154.53 | 366 | 0.945 | 0.939 | 0.073 | Fit virtually unchanged (ΔCFI < 0.001; ΔAIC = +5.24); no fit-based justification for item removal |
| D. Bifactor Model Fit Indices (Primary Model) | 699.11 | 348 | 0.958 | 0.951 | 0.050 | Primary model; satisfactory fit |

*Notes: *Model A was estimated under the original item-level specification and is retained to demonstrate the necessity of the hierarchical second-order structure; its Process and Outcome measurement structure is identical to that of the primary model. Models B and C share identical degrees of freedom because Item 1.1.2 enters the model only through the Human Resources composite score; their comparison is therefore descriptive rather than a nested chi-square difference test. Modification indices involving structural components are not listed, as the formative structural dimension has no covarying factor residuals; all listed MIs were rejected to preserve the a priori theoretical structure. The primary model in the revised manuscript is the Bifactor specification (fit indices reported in primary manuscript Table 3; omega estimates in Table 2); Models A, B, and C in this table are retained as supplementary comparison models to demonstrate the necessity of the Bifactor re-specification. Model B's second-order HTMT = 1.06 and first-order HTMT > 0.90 for several pairs are the empirical triggers that motivated the Bifactor re-specification.*

**Table S4. Standardized factor loadings, MLR robust standard errors, and 95% Wald confidence intervals for the Bifactor model (N = 410)**

| **Dimension /Sub-dimension** | **Item Code** | **Item Content (Abbreviated)** | **Specific Factor** | **General Factor λ** | **Specific Factor λ** | **R²** | **Residual Variance** | **MLR Robust SE** | **General 95% Wald CI (Lower)** | **General 95% Wald CI (Upper)** | **Specific MLR Robust SE** | **Specific 95% Wald CI (Lower)** | **Specific 95% Wald CI (Upper)** |
| --- | --- | --- | --- | --- | --- | --- | --- | --- | --- | --- | --- | --- | --- |
| **Process dimension** |  |  |  |  |  |  |  |  |  |  |  |  |  |
| Nursing Assessment | 2.1.1 | Correct screening tool use for new stroke patients | Assessment | 0.744 | 0.289 | 0.637 | 0.363 | 0.048 | 0.651 | 0.837 | 0.05 | 0.191 | 0.387 |
|  | 2.1.2 | MDT correctly co-assesses dysphagia | Assessment | 0.695 | 0.604 | 0.848 | 0.152 | 0.045 | 0.607 | 0.783 | 0.069 | 0.468 | 0.74 |
|  | 2.1.3 | Correct nutritional risk screening tool use | Assessment | 0.745 | 0.392 | 0.709 | 0.291 | 0.044 | 0.659 | 0.831 | 0.053 | 0.288 | 0.496 |
|  | 2.1.4 | Correct oral hygiene assessment & care solution selection | Assessment | 0.804 | 0.18 | 0.679 | 0.321 | 0.045 | 0.716 | 0.892 | 0.05 | 0.082 | 0.278 |
| Nursing Practice | 2.2.1 | Correct feeding route selection | Practice | 0.776 | 0.252 | 0.666 | 0.334 | 0.044 | 0.691 | 0.862 | 0.06 | 0.134 | 0.369 |
|  | 2.2.2 | Correct feeding posture selection | Practice | 0.782 | 0.375 | 0.752 | 0.248 | 0.047 | 0.689 | 0.875 | 0.055 | 0.266 | 0.484 |
|  | 2.2.3 | Correct judgment of feeding pace | Practice | 0.81 | 0.346 | 0.776 | 0.224 | 0.048 | 0.716 | 0.904 | 0.058 | 0.232 | 0.46 |
|  | 2.2.4 | Correct guidance on food consistency | Practice | 0.839 | 0.432 | 0.891 | 0.109 | 0.049 | 0.742 | 0.936 | 0.042 | 0.351 | 0.514 |
|  | 2.2.5 | Correct judgment of feeding utensils | Practice | 0.842 | 0.397 | 0.867 | 0.133 | 0.047 | 0.749 | 0.935 | 0.047 | 0.305 | 0.489 |
|  | 2.2.6 | Correct oral care procedure | Practice | 0.818 | 0.315 | 0.768 | 0.232 | 0.048 | 0.724 | 0.912 | 0.055 | 0.206 | 0.423 |
|  | 2.2.7 | Personalized feeding plan effectively implemented | Practice | 0.868 | 0.131 | 0.77 | 0.23 | 0.047 | 0.776 | 0.96 | 0.045 | 0.043 | 0.219 |
| Health Education | 2.3.1 | Comprehensive education for inpatients with dysphagia | Education | 0.854 | 0.148 | 0.751 | 0.249 | 0.048 | 0.759 | 0.948 | 0.077 | -0.003 | 0.299 |
|  | 2.3.2 | Dysphagia knowledge education achieves expected outcomes | Education | 0.865 | 0.201 | 0.789 | 0.211 | 0.048 | 0.772 | 0.959 | 0.073 | 0.058 | 0.345 |
|  | 2.3.3 | Patients master rehabilitation techniques post-education | Education | 0.886 | 0.317 | 0.886 | 0.114 | 0.048 | 0.792 | 0.98 | 0.078 | 0.164 | 0.471 |
|  | 2.3.4 | Detailed discharge rehabilitation guidance provided | Education | 0.88 | 0.168 | 0.802 | 0.198 | 0.047 | 0.788 | 0.972 | 0.052 | 0.066 | 0.27 |
| Follow-up Management | 2.4.1 | Timely patient follow-up completion | Follow-up | 0.858 | 0.341 | 0.853 | 0.147 | 0.048 | 0.764 | 0.952 | 0.09 | 0.165 | 0.518 |
|  | 2.4.2 | Accurate follow-up documentation | Follow-up | 0.876 | 0.336 | 0.881 | 0.119 | 0.048 | 0.783 | 0.97 | 0.086 | 0.167 | 0.505 |
|  | 2.4.3 | High patient follow-up appointment adherence | Follow-up | 0.842 | 0.126 | 0.724 | 0.276 | 0.048 | 0.747 | 0.936 | 0.054 | 0.019 | 0.232 |
| **Outcome dimension** |  |  |  |  |  |  |  |  |  |  |  |  |  |
| Nurse Outcomes | 3.1.1 | Strong nurse dysphagia knowledge | Nurse Outcomes | 0.849 | 0.287 | 0.804 | 0.196 | 0.046 | 0.759 | 0.939 | 0.065 | 0.160 | 0.415 |
|  | 3.1.2 | Proficient nurse dysphagia skills | Nurse Outcomes | 0.874 | 0.358 | 0.891 | 0.109 | 0.047 | 0.781 | 0.966 | 0.051 | 0.257 | 0.458 |
|  | 3.1.3 | High physician satisfaction with nursing care | Nurse Outcomes | 0.871 | 0.329 | 0.867 | 0.133 | 0.048 | 0.778 | 0.965 | 0.061 | 0.209 | 0.448 |
| Patient Outcomes | 3.2.1 | Near-absence of dysphagia-related adverse events (per EMR) | Patient Outcomes | 0.786 | 0.247 | 0.679 | 0.321 | 0.049 | 0.689 | 0.883 | 0.055 | 0.139 | 0.355 |
|  | 3.2.2 | High patient/family satisfaction with rehabilitation care | Patient Outcomes | 0.835 | 0.351 | 0.82 | 0.18 | 0.048 | 0.742 | 0.928 | 0.051 | 0.252 | 0.451 |
|  | 3.2.3 | High patient compliance with rehabilitation therapy | Patient Outcomes | 0.854 | 0.314 | 0.827 | 0.173 | 0.047 | 0.761 | 0.946 | 0.048 | 0.220 | 0.407 |
|  | 3.2.4 | Near-absence of aspiration pneumonia | Patient Outcomes | 0.773 | 0.363 | 0.729 | 0.271 | 0.047 | 0.68 | 0.865 | 0.065 | 0.236 | 0.49 |
|  | 3.2.5 | Significant improvement in swallowing function | Patient Outcomes | 0.873 | 0.302 | 0.853 | 0.147 | 0.045 | 0.784 | 0.962 | 0.040 | 0.223 | 0.381 |

*Notes: N = 410. All 26 reflective items simultaneously load on the General Factor (NMC-PSD General Capacity) and one orthogonal Specific Factor. Standardized loadings (λ) are fully standardized (std.all). Standard errors are MLR robust standard errors (Huber-White sandwich estimator). Ninety-five percent confidence intervals are Wald intervals computed as estimate ± 1.96 × MLR robust SE. All general-factor loadings are statistically significant at p < .001. Specific-factor loadings are reported for completeness; their comparatively lower magnitude reflects the dominant general factor. Because Bollen–Stine bootstrap resampling (B = 2,000) produced pervasive convergence failures and Heywood cases in the orthogonal bifactor specification, bootstrap standard errors were uninterpretable and are not reported. All inferential statistics were therefore based on MLR (maximum likelihood with robust standard errors; Satorra–Bentler scaled test statistics), which provides standard errors robust to multivariate non-normality without resampling. MLR and bootstrap standard errors are distinct methods; no claim of equivalence is made (Kline, 2016). Model fit: χ²(348) = 699.11, CFI = 0.958, TLI = 0.951, RMSEA = 0.050 (90% CI: 0.045–0.054), SRMR = 0.027.*

**Clustering diagnostics and conservative robustness checks for the NMC-PSD Scale (N = 410)**

Because hospital and unit identifiers were not recorded in the anonymous survey, clustering was assessed at the two grouping levels observable in the data (province and city). In addition, an assumption-based scenario analysis was conducted as a conservative robustness check for the unobservable unit level; this analysis assumes, rather than estimates, the clustering parameters and does not constitute cluster-adjusted estimation.

**Table S5.1. Province-level clustering diagnostics (*N* = 410; 12 provinces; adjusted mean cluster size n̄* = 23.16)**

| **Variable** | **σ² (between)** | **σ² (within)** | **ICC(1), REML** | **ICC(1), ANOVA†** | **Design effect‡** | **Effective N** |
| --- | --- | --- | --- | --- | --- | --- |
| Total score | 4.42 | 501.92 | 0.009 | 0.037 | 1.19 | 344 |
| Structure dimension | 0.00 | 33.37 | 0.000 | −0.001 | 1.00 | 410 |
| Process dimension | 1.54 | 154.84 | 0.010 | 0.046 | 1.22 | 337 |
| Outcome dimension | 0.39 | 31.92 | 0.012 | 0.034 | 1.27 | 323 |

**Table S5.2. City-level clustering diagnostics (*N* = 410; 23 cities; adjusted mean cluster size n̄* = 11.77)**

| **Variable** | **ICC(1), REML** | **Design effect** | **Effective N** |
| --- | --- | --- | --- |
| Total score | 0.004 | 1.05 | 391 |
| Structure dimension | <0.001 | 1.00 | 410 |
| Process dimension | 0.007 | 1.07 | 382 |
| Outcome dimension | 0.008 | 1.09 | 377 |

**Table S5.3. Assumption-based scenario analysis for the unobservable unit level (conservative robustness check)**

| **Scenario** | **Assumed ICC(1)** | **Assumed mean cluster size** | **Design effect** | **Effective N** | **Adjusted χ²/df** |
| --- | --- | --- | --- | --- | --- |
| Observed maximum (province, ANOVA estimator) | 0.046 | 23.16 | 2.01 | 204 | 1.56 |
| Moderate | 0.05 | 10 | 1.45 | 283 | 2.17 |
| Strong | 0.10 | 10 | 1.90 | 216 | 1.65 |
| Highly conservative | 0.15 | 10 | 2.35 | 174 | 1.34 |

**Table S5.4. Standard error inflation check at the largest design effect (2.35)**

| **Parameter** | **Unadjusted C.R.** | **Inflation factor (√2.35)** | **Adjusted C.R.** | **Adjusted p** |
| --- | --- | --- | --- | --- |
| Minimum standardized loading (Item 2.1.2, 0.695) | 15.44 | 1.53 | 10.08 | <0.01 |

All remaining standardized loadings (range 0.81–0.94) had larger unadjusted critical ratios and therefore also remained statistically significant after inflation.

*Notes. ICC(1) = σ²between / (σ²between + σ²within), estimated from random-intercept mixed models fitted by restricted maximum likelihood (REML). † The ANOVA method-of-moments estimator, (MSB − MSW) / (MSB + (n̄* − 1) × MSW), is reported as a sensitivity analysis given the small number of clusters and unequal cluster sizes. ‡ Design effect = 1 + (n̄* − 1) × ICC(1), where n̄* = (N − Σni²/N) / (k − 1) adjusts for unequal cluster sizes. Scenario ICC values (0.05–0.15) span and exceed the range commonly reported for attitudinal and organizational-perception surveys in health settings (Gulliford, Ukoumunne, & Chinn, 1999). Chi-square scaling by design effect follows the approach for complex-sample structural equation modeling (Muthén & Satorra, 1995); because ignoring clustering overestimates the chi-square statistic, adjusted values represent conservative bounds.*

**References**

Gulliford, M. C., Ukoumunne, O. C., & Chinn, S. (1999). Components of variance and intraclass correlations for the design of community-based surveys and intervention studies: Data from the Health Survey for England 1994. American Journal of Epidemiology, 149(9), 876–883.

Muthén, B., & Satorra, A. (1995). Complex sample data in structural equation modeling. Sociological Methodology, 25, 267–316.
