# Supplementary material for: Development and Psychometric Evaluation of the Nursing Management Capacity Scale for Poststroke Dysphagia Based on the Donabedian Model
Source: J Nurs Manag. 2026 Sep 20;2026:9248618. doi: 10.1155/jonm/9248618 (PMC13591012; doi:10.1155/jonm/9248618)
Supplement: Supplementary file 1 [file JONM-2026-9248618-s001.zip › Supplementary File 3_Two-Round Delphi Consultation.docx]

**Supplementary File 3. Two-Round Delphi Consultation: Procedure, Round-Specific Changes, and Content Validity Indices**

## 1. Panel and Administration

The consultation panel comprised ten healthcare experts (2 nursing administrators, 2 clinical nurses, 3 clinical physicians, and 3 rehabilitation therapists), selected by purposive sampling according to the criteria reported in Section 2.2.1 of the main text. The same panel completed both rounds anonymously via electronic questionnaires (Round 1: January 2025; Round 2: February 2025). Experts were blinded to each other's identities and ratings, and no discussion was permitted. All ten experts returned completed questionnaires in both rounds (response rate: 100%). Panel authority was quantified using the expert authority coefficient, Cr = (Cs + Ca)/2, where Cs is the familiarity coefficient and Ca the judgment-basis coefficient (theoretical analysis, clinical experience, literature, and intuition, weighted by self-rated impact). The mean Cr was 0.88 (range: 0.70–0.95), exceeding the conventional acceptability threshold of 0.70.

## 2. Round 1 (January 2025)

Experts rated the importance of each of the 35 items on a 4-point scale and provided item-level written comments. Item importance means ranged from 2.30 to 3.80, with coefficients of variation (CV) of 0.11–0.32. Kendall's coefficient of concordance was 0.257 (χ² = 87.30, df = 34, p < 0.001), and the intraclass correlation coefficient for the average of the ten raters (ICC[2,10], two-way random effects, absolute agreement) was 0.684 (single-rater ICC[2,1] = 0.178). Because the pre-specified consensus criteria (Section 4) were not met, a second round was conducted.

## 3. Inter-Round Revisions and Feedback Presentation

Written feedback was anonymized and collated by the research team into 11 thematic categories (Table S6b). The collated summary, the revised questionnaire, and a cover letter documenting adopted, modified, and non-adopted suggestions (with rationales) were returned to the same panel. Adopted revisions included: defined rating anchors; explicit reverse-scoring rules for reverse-worded items; replacement of absolute ("100%") thresholds with unit-defined standards; a not-applicable option for items with legitimate non-response; operational definitions and observation windows for outcome items; and permission of equivalent validated assessment tools. Item splitting was not adopted in order to preserve one-to-one fidelity with the source indicator framework. In addition, the item pool was re-verified against the source checklist [37]: one management-system item not belonging to the original 35-indicator framework was removed, and the patient/family satisfaction indicator (source indicator III-2-2) was restored, so that the final 35 items correspond one-to-one with the source indicator system.

## 4. Round 2 (February 2025) and Termination

Experts re-rated each item's relevance (4-point scale: 1 = irrelevant to 4 = highly relevant) and clarity (3-point scale). Consensus was defined a priori as: (i) I-CVI ≥ 0.78 for every item; (ii) S-CVI/Ave ≥ 0.90; and (iii) CV < 0.25 for all items. All criteria were met: item relevance means ranged from 3.20 to 4.00 (CV: 0.00–0.25), I-CVI ranged from 0.80 to 1.00, S-CVI/Ave was 0.93, and modified kappa ranged from 0.79 to 1.00 (Table S6c). The consultation was therefore terminated after two rounds. Kendall's W in Round 2 was 0.128 (χ² = 43.42, df = 34, p = 0.129); because relevance ratings converged toward the top of the scale, variance-based concordance indices (W and ICC) lose discriminatory power in Round 2, and consensus was therefore indexed by I-CVI and CV, consistent with Delphi and content-validation conventions. A round-by-round summary is provided in Table S6a.

Table S6a. Summary of the two-round expert consultation (same panel, n = 10).

| **Item** | **Round 1 (January 2025)** | **Round 2 (February 2025)** |
| --- | --- | --- |
| Experts invited / completed | 10 / 10 | 10 / 10 |
| Response rate | 100% | 100% |
| Task | Importance rating + item-level written comments | Relevance and clarity re-rating |
| Scale | 4-point importance | 4-point relevance; 3-point clarity |
| Item means (range) | 2.30–3.80 | 3.20–4.00 |
| Coefficient of variation (range) | 0.11–0.32 | 0.00–0.25 |
| Kendall's W | 0.257 (χ² = 87.30, df = 34, p < 0.001) | 0.128 (χ² = 43.42, df = 34, p = 0.129) |
| ICC(2,10) | 0.684 | Not informative (ceiling concentration) |
| Consensus criteria met | No (CV up to 0.32; revisions required) | Yes (I-CVI ≥ 0.78 all items; S-CVI/Ave = 0.93; CV < 0.25 all items) |
| Panel authority, mean Cr (range) | 0.88 (0.70–0.95), assessed once for the standing panel | Same panel |

Table S6b. Round 1 expert feedback and actions taken before Round 2.

| **No.** | **Feedback theme** | **Items** | **Summary of expert concern** | **Action taken** |
| --- | --- | --- | --- | --- |
| 1 | Undefined rating anchors | All | No clinical meaning defined for scale points; scoring purely subjective | Adopted — standardized 4-point rating anchors defined in the Round 2 instructions; final Likert response scale anchored uniformly |
| 2 | Reverse-scoring rules | 34, 35 | Reverse items flagged but conversion rule unspecified | Adopted —the entry has been rewritten in the forward direction and does not require reverse encoding |
| 3 | Compound items | 11, 12, 27 | Multiple assessment points within one item | Not adopted (documented) — items retained unsplit to preserve one-to-one fidelity with the source framework; rationale stated in the Round 2 cover letter |
| 4 | Absolute thresholds ("100%") | 21, 22, 25, 26, 28 | Clinically unattainable; ceiling compression of scores | Adopted — replaced with unit-defined standards (meeting unit-specified standards) |
| 5 | Binary attribute on graded scale | 7 | Yes/no attribute forced onto a graded scale | Partially adopted — retained in Round 2; reworded as a graded adequacy statement ("Complete & functional emergency equipment") in the final Likert conversion |
| 6 | Mixed unit of assessment | 1–10 vs 15–28 | Unit-level resources vs patient-level behaviors mixed | Adopted — all items standardized to unit-referent, nurse-perceived adequacy statements in the final scale |
| 7 | Missing not-applicable option | 5, 25, 26, 28 | Legitimate non-applicability penalized as low score | Adopted — not-applicable annotation added (e.g., item 5) |
| 8 | Undefined outcome threshold | 33 | "Significant improvement" lacked an operational definition | Adopted — defined as ≥1-grade improvement on the Kubota water swallow test or V-VST improvement |
| 9 | Undefined observation window | 35 | Incidence window for aspiration pneumonia unspecified | Adopted — window restricted to the hospitalization period |
| 10 | Tool specificity | 13 | NRS2002 not universally used across units | Adopted — equivalent validated assessment tools explicitly permitted |
| 11 | Questionnaire formatting | All | Duplicated and mixed table headers | Adopted — Round 2 questionnaire reformatted with a single standardized header |
| 12 | Item-pool re-verification | Management-system item; patient/family satisfaction item | Item pool re-checked against the source checklist [37] | Adopted — one management-system item not present in the source framework removed; the patient/family satisfaction indicator (source indicator III-2-2) restored; final 35 items correspond one-to-one to Guo et al. [37]. |

Table S6c. Content Validity Indices for Each Item (N = 10 Experts).

| **Item** | **Dimension** | **I-CVI** | **A** | **Pc** | **k*** | **Decision** |
| --- | --- | --- | --- | --- | --- | --- |
| 1.1.1 | Structure | 1 | 10 | 0.001 | 1 | Retained |
| 1.1.2 | Structure | 1 | 10 | 0.001 | 1 | Retained |
| 1.1.3 | Structure | 1 | 10 | 0.001 | 1 | Retained |
| 1.1.4 | Structure | 1 | 10 | 0.001 | 1 | Retained |
| 1.1.5 | Structure | 1 | 10 | 0.001 | 1 | Retained |
| 1.2.1 | Structure | 1 | 10 | 0.001 | 1 | Retained |
| 1.2.2 | Structure | 0.9 | 9 | 0.0098 | 0.9 | Retained |
| 1.3.1 | Structure | 0.9 | 9 | 0.0098 | 0.9 | Retained |
| 1.3.2 | Structure | 0.8 | 8 | 0.0439 | 0.79 | Retained |
| 2.1.1 | Process | 0.9 | 9 | 0.0098 | 0.9 | Retained |
| 2.1.2 | Process | 0.9 | 9 | 0.0098 | 0.9 | Retained |
| 2.1.3 | Process | 1 | 10 | 0.001 | 1 | Retained |
| 2.1.4 | Process | 1 | 10 | 0.001 | 1 | Retained |
| 2.2.1 | Process | 1 | 10 | 0.001 | 1 | Retained |
| 2.2.2 | Process | 0.9 | 9 | 0.0098 | 0.9 | Retained |
| 2.2.3 | Process | 1 | 10 | 0.001 | 1 | Retained |
| 2.2.4 | Process | 0.8 | 8 | 0.0439 | 0.79 | Retained |
| 2.2.5 | Process | 1 | 10 | 0.001 | 1 | Retained |
| 2.2.6 | Process | 0.8 | 8 | 0.0439 | 0.79 | Retained |
| 2.2.7 | Process | 0.9 | 9 | 0.0098 | 0.9 | Retained |
| 2.3.1 | Process | 0.9 | 9 | 0.0098 | 0.9 | Retained |
| 2.3.2 | Process | 0.9 | 9 | 0.0098 | 0.9 | Retained |
| 2.3.3 | Process | 1 | 10 | 0.001 | 1 | Retained |
| 2.3.4 | Process | 1 | 10 | 0.001 | 1 | Retained |
| 2.4.1 | Process | 0.9 | 9 | 0.0098 | 0.9 | Retained |
| 2.4.2 | Process | 0.9 | 9 | 0.0098 | 0.9 | Retained |
| 2.4.3 | Process | 0.9 | 9 | 0.0098 | 0.9 | Retained |
| 3.1.1 | Outcome | 0.8 | 8 | 0.0439 | 0.79 | Retained |
| 3.1.2 | Outcome | 1 | 10 | 0.001 | 1 | Retained |
| 3.1.3 | Outcome | 0.9 | 9 | 0.0098 | 0.9 | Retained |
| 3.2.1 | Outcome | 0.8 | 8 | 0.0439 | 0.79 | Retained |
| 3.2.2 | Outcome | 0.9 | 9 | 0.0098 | 0.9 | Retained |
| 3.2.3 | Outcome | 0.9 | 9 | 0.0098 | 0.9 | Retained |
| 3.2.4 | Outcome | 1 | 10 | 0.001 | 1 | Retained |
| 3.2.5 | Outcome | 0.9 | 9 | 0.0098 | 0.9 | Retained |
| S-CVI/Ave |  | 0.929 |  |  |  | Satisfactory |

Note: I-CVI = item-level content validity index; S-CVI/Ave = scale-level content validity index (average method); k* = Polit-Beck modified kappa; A = number of experts rating the item as relevant (score ≥ 3 on a 4-point scale). Thresholds: I-CVI ≥ 0.78, S-CVI/Ave ≥ 0.90, k* > 0.74. I-CVI, A, Pc, and k* were computed from Round 2 relevance ratings.
