# Supplementary material for: Development and Psychometric Evaluation of the Nursing Management Capacity Scale for Poststroke Dysphagia Based on the Donabedian Model
Source: J Nurs Manag. 2026 Sep 20;2026:9248618. doi: 10.1155/jonm/9248618 (PMC13591012; doi:10.1155/jonm/9248618)
Supplement: Supplementary file 1 [file JONM-2026-9248618-s001.zip › Supplementary File 4_Reproducible lavaan Model Syntax and Output for the Bifactor Analysis.docx]

**Supplementary File 4**

**Reproducible lavaan Model Syntax and Output for the Bifactor Analysis**

This supplementary file provides all materials necessary to reproduce the bifactor confirmatory factor analysis (CFA) and formative–bifactor hybrid model reported in the manuscript (Methods, Section 2.3.5; Results, Sections 3.2.4 and 3.2.5; Table 2; Table 3). All R code, model output, and result tables are consolidated here in a single document for ease of review.

**CONTENTS**

(1) Reproducible R syntax (NMC_PSD_Bifactor_MLR_Analysis_Clean.R) — Section 1

(2) Full lavaan output for the primary MLR model (Lavaan_Summary.txt) — Section 2

(3) Model fit indices (Table_Fit_Indices.csv) — Section 3, Table 3.1

(4) Bifactor indices, ECV/omegaH/omegaHS (Table_Bifactor_Indices.csv) — Section 3, Table 3.2

(5) Formative structural regression weights (Table_Formative_Regression.csv) — Section 3, Table 3.3

(6) Standardized factor loadings with MLR robust SEs (Table_S4_Loadings_MLR.csv) — Section 3, Table 3.4

# Section 1. Reproducible R Syntax (NMC_PSD_Bifactor_MLR_Analysis_Clean.R)

The following R script implements the formative–bifactor hybrid model estimated with robust maximum likelihood (MLR). The script includes data loading and validation (Q1–Q35, Likert 1–5), formative composite computation (Human Resources, Facilities & Equipment, Management Systems), model specification with an orthogonal general factor and six specific factors, MLR estimation with Satorra–Bentler scaled test statistics, the Bollen–Stine bootstrap attempt (2000 samples) with convergence-failure handling, standardized factor loadings extraction with robust SEs, Wald 95% confidence intervals (unstandardized estimates), model fit indices extraction, bifactor indices computation (ECV, ωH, ωHS) via the BifactorIndicesCalculator package, and formative regression weights extraction.

*Software requirements: R ≥ 4.0; lavaan ≥ 0.6-19; BifactorIndicesCalculator.*

```
# ============================================================
# NMC-PSD Scale: Formative-Bifactor Hybrid CFA with MLR
# Corresponding manuscript section: 2.3.5 Confirmatory Factor Analysis
#
# Core contents:
#   1. MLR (robust maximum likelihood) estimation for bifactor model
#   2. Bollen-Stine bootstrap attempt (2000 samples)
#   3. Fallback to MLR robust standard errors + Wald 95% CI when bootstrap fails
#   4. Bifactor Indices computation (ECV, omegaH, omegaHS)
#   5. Standardized loadings output (Supplementary File 2, Table S4 format)
#
# Software environment: R >= 4.0, lavaan >= 0.6-19, BifactorIndicesCalculator
# ============================================================

# ------------------------------------------------------------
# 0. Package loading
# ------------------------------------------------------------
cat("=== NMC-PSD Bifactor CFA Analysis ===\n")
cat("Loading required packages...\n")

if (!requireNamespace("lavaan", quietly = TRUE)) {
  install.packages("lavaan", repos = "https://cloud.r-project.org/")
}
if (!requireNamespace("BifactorIndicesCalculator", quietly = TRUE)) {
  install.packages("BifactorIndicesCalculator", repos = "https://cloud.r-project.org/")
}

library(lavaan)
library(BifactorIndicesCalculator)

cat("Packages loaded.\n\n")

# ------------------------------------------------------------
# 1. Data reading (real data)
# ------------------------------------------------------------
# [User data path]
data_path <- "D:/Documents/Desktop/Bifactor/data410.csv"

data <- read.csv(data_path, header = TRUE, stringsAsFactors = FALSE)

# Check data dimensions
N <- nrow(data)
n_items <- ncol(data)
cat(sprintf("Data loaded from: %s\n", data_path))
cat(sprintf("Sample size: N = %d\n", N))
cat(sprintf("Number of items: %d\n\n", n_items))

# Check column names are Q1-Q35
expected_names <- paste0("Q", 1:35)
if (!all(expected_names %in% names(data))) {
  stop("Column names do not match expected Q1-Q35. Please check data file.")
}

# Check data range (Likert 1-5)
for (col in expected_names) {
  if (any(data[[col]] < 1 | data[[col]] > 5, na.rm = TRUE)) {
    cat(sprintf("Warning: %s contains values outside 1-5 range\n", col))
  }
}

# ------------------------------------------------------------
# 2. Formative Composite Computation (Observed Single-Indicator)
# ------------------------------------------------------------
# Structure Dimension (9 items, formative):
#   Q1-Q5  : 1.1 Human Resources
#   Q6-Q7  : 1.2 Facilities & Equipment
#   Q8-Q9  : 1.3 Management Systems

# Human Resources (5 items)
data$HR <- rowMeans(data[, c("Q1", "Q2", "Q3", "Q4", "Q5")], na.rm = TRUE)
# Facilities and Equipment (2 items)
data$FE <- rowMeans(data[, c("Q6", "Q7")], na.rm = TRUE)
# Management Systems (2 items)
data$MS <- rowMeans(data[, c("Q8", "Q9")], na.rm = TRUE)

cat("Formative composites computed:\n")
cat("  HR (Q1-Q5):  Human Resources\n")
cat("  FE (Q6-Q7):  Facilities & Equipment\n")
cat("  MS (Q8-Q9):  Management Systems\n\n")

# ------------------------------------------------------------
# 3. Model Specification: Formative-Bifactor Hybrid
# ------------------------------------------------------------
# Reflective items mapping:
#   Q10-Q13 : 2.1 Assessment (4 items)
#   Q14-Q20 : 2.2 Practice (7 items)
#   Q21-Q24 : 2.3 Education (4 items)
#   Q25-Q27 : 2.4 Follow-up (3 items)
#   Q28-Q30 : 3.1 Nurse-related Outcomes (3 items)
#   Q31-Q35 : 3.2 Patient-related Outcomes (5 items)

model_full <- '
  # === General Factor (26 reflective Process/Outcome items: Q10-Q35) ===
  gen =~ Q10 + Q11 + Q12 + Q13 +
         Q14 + Q15 + Q16 + Q17 + Q18 + Q19 + Q20 +
         Q21 + Q22 + Q23 + Q24 +
         Q25 + Q26 + Q27 +
         Q28 + Q29 + Q30 +
         Q31 + Q32 + Q33 + Q34 + Q35

  # === Six Orthogonal Specific Factors ===
  assess   =~ Q10 + Q11 + Q12 + Q13
  practice =~ Q14 + Q15 + Q16 + Q17 + Q18 + Q19 + Q20
  educ     =~ Q21 + Q22 + Q23 + Q24
  follow   =~ Q25 + Q26 + Q27
  nurse_out=~ Q28 + Q29 + Q30
  patient_out=~ Q31 + Q32 + Q33 + Q34 + Q35

  # === Orthogonality: General vs. All Specific Factors ===
  gen ~~ 0*assess + 0*practice + 0*educ + 0*follow + 0*nurse_out + 0*patient_out

  # === Orthogonality: Among Specific Factors ===
  assess ~~ 0*practice + 0*educ + 0*follow + 0*nurse_out + 0*patient_out
  practice ~~ 0*educ + 0*follow + 0*nurse_out + 0*patient_out
  educ ~~ 0*follow + 0*nurse_out + 0*patient_out
  follow ~~ 0*nurse_out + 0*patient_out
  nurse_out ~~ 0*patient_out

  # === Formative Structural Dimension Predicting General Factor ===
  gen ~ HR + FE + MS
'

cat("Model specification defined.\n")
cat("  - 26 reflective items (Q10-Q35) on general + 6 specific factors\n")
cat("  - 9 formative structural items (Q1-Q9) -> 3 composites -> gen\n")
cat("  - All specific factors orthogonal to general and to each other\n\n")

# ------------------------------------------------------------
# 4. Model Fitting: MLR Estimation (Primary Estimator)
# ------------------------------------------------------------
cat("=== Fitting Primary Model with MLR ===\n")
fit_mlr <- cfa(
  model = model_full,
  data  = data,
  estimator = "MLR",      # Robust ML
  std.lv = TRUE,          # Fix latent variable variance to 1
  missing = "fiml"        # Full Information ML
)

cat("MLR model fitted successfully.\n\n")

# ------------------------------------------------------------
# 5. Bollen-Stine Bootstrap Attempt
# ------------------------------------------------------------
cat("=== Attempting Bollen-Stine Bootstrap (2000 samples) ===\n")
cat("NOTE: In the actual study, this step produced pervasive\n")
cat("      convergence failures and Heywood cases.\n\n")

fit_bs <- tryCatch({
  cfa(
    model = model_full,
    data  = data,
    estimator = "MLR",
    test = "bollen.stine",
    se = "bootstrap",
    bootstrap = 2000,
    std.lv = TRUE
  )
}, warning = function(w) {
  cat("Bootstrap produced warnings:", conditionMessage(w), "\n")
  tryCatch(
    cfa(model = model_full, data = data, estimator = "MLR",
        test = "bollen.stine", se = "bootstrap", bootstrap = 2000, std.lv = TRUE),
    error = function(e) NULL
  )
}, error = function(e) {
  cat("Bootstrap FAILED with error:", conditionMessage(e), "\n")
  cat("--------------------------------------------------------\n")
  cat("INTERPRETATION (for manuscript Methods section):\n")
  cat("  The orthogonal bifactor resampling produced pervasive\n")
  cat("  convergence failures and Heywood cases, rendering\n")
  cat("  bootstrap standard errors and percentile confidence\n")
  cat("  intervals uninterpretable. Consequently, all inferential\n")
  cat("  statistics are based on MLR robust standard errors, which\n")
  cat("  provide consistent parameter estimates under multivariate\n")
  cat("  non-normality when bootstrap resampling fails due to\n")
  cat("  pervasive convergence issues (Kline, 2016; Rosseel, 2012).\n")
  cat("--------------------------------------------------------\n\n")
  NULL
})

if (!is.null(fit_bs)) {
  cat("Bootstrap completed.\n\n")
} else {
  cat("Bootstrap unavailable; proceeding with MLR robust SE.\n\n")
}

# ------------------------------------------------------------
# 6. Extract MLR Results: Standardized Loadings, Robust SE, z, 95% Wald CI
# ------------------------------------------------------------
cat("=== Extracting MLR Robust Results ===\n")

pe <- parameterEstimates(fit_mlr, standardized = TRUE, ci = TRUE, level = 0.95)

# Filter standardized loadings (latent -> observed, i.e., op == "=~")
loadings_table <- pe[pe$op == "=~" & pe$lhs %in% c("gen", "assess", "practice", "educ", "follow", "nurse_out", "patient_out"), ]

# Reorder: General factor first, then specific factors
factor_order <- c("gen", "assess", "practice", "educ", "follow", "nurse_out", "patient_out")
loadings_table$lhs <- factor(loadings_table$lhs, levels = factor_order)
loadings_table <- loadings_table[order(loadings_table$lhs, loadings_table$rhs), ]

# Rename columns to match manuscript Table S4 format
names(loadings_table)[names(loadings_table) == "lhs"] <- "Factor"
names(loadings_table)[names(loadings_table) == "rhs"] <- "Item"
names(loadings_table)[names(loadings_table) == "est"] <- "Unstd_Est"
names(loadings_table)[names(loadings_table) == "se"] <- "Robust_SE"
names(loadings_table)[names(loadings_table) == "z"] <- "z_value"
names(loadings_table)[names(loadings_table) == "pvalue"] <- "p_value"
names(loadings_table)[names(loadings_table) == "ci.lower"] <- "Unstd_CI_95_Lower"
names(loadings_table)[names(loadings_table) == "ci.upper"] <- "Unstd_CI_95_Upper"
names(loadings_table)[names(loadings_table) == "std.all"] <- "Std_Loading"

# Select key columns for output
output_cols <- c("Factor", "Item", "Std_Loading", "Robust_SE", "z_value", "p_value", "Unstd_CI_95_Lower", "Unstd_CI_95_Upper")
loadings_out <- loadings_table[, output_cols]

# Format p-value
loadings_out$p_value <- ifelse(loadings_out$p_value < 0.001, "<.001",
                               sprintf("%.3f", loadings_out$p_value))

cat("\nStandardized Factor Loadings (MLR Robust SE, 95% Wald CI on unstd estimates):\n")
print(loadings_out, row.names = FALSE)

# Check General factor loadings range
gen_loadings <- loadings_out[loadings_out$Factor == "gen", "Std_Loading"]
cat(sprintf("\nGeneral factor loadings range: %.2f - %.2f\n",
            min(gen_loadings, na.rm = TRUE), max(gen_loadings, na.rm = TRUE)))

# Check Significance
all_sig <- all(loadings_table$pvalue[loadings_table$Factor == "gen"] < 0.001, na.rm = TRUE)
cat(sprintf("All general factor loadings significant at p < .001: %s\n\n", all_sig))

# ------------------------------------------------------------
# 7. Fit Indices Extraction
# ------------------------------------------------------------
cat("=== Model Fit Indices (MLR Scaled) ===\n")
fit_idx <- fitMeasures(fit_mlr, c(
  "chisq.scaled", "df", "pvalue.scaled",
  "cfi.scaled", "tli.scaled",
  "rmsea.scaled", "rmsea.ci.lower.scaled", "rmsea.ci.upper.scaled",
  "srmr"
))

fit_summary <- data.frame(
  Index = c("Chi2 (scaled)", "df", "p-value (scaled)",
            "CFI (scaled)", "TLI (scaled)",
            "RMSEA (scaled)", "RMSEA 90% CI Lower", "RMSEA 90% CI Upper",
            "SRMR"),
  Value = c(
    sprintf("%.2f", fit_idx["chisq.scaled"]),
    sprintf("%.0f", fit_idx["df"]),
    sprintf("%.3f", fit_idx["pvalue.scaled"]),
    sprintf("%.3f", fit_idx["cfi.scaled"]),
    sprintf("%.3f", fit_idx["tli.scaled"]),
    sprintf("%.3f", fit_idx["rmsea.scaled"]),
    sprintf("%.3f", fit_idx["rmsea.ci.lower.scaled"]),
    sprintf("%.3f", fit_idx["rmsea.ci.upper.scaled"]),
    sprintf("%.3f", fit_idx["srmr"])
  )
)
print(fit_summary, row.names = FALSE)
cat("\n")

# ------------------------------------------------------------
# 8. Bifactor Indices Computation (ECV, omegaH, omegaHS)
# ------------------------------------------------------------
# Note: BifactorIndicesCalculator requires a pure measurement model
# (no exogenous predictors). Therefore, we fit the 26 reflective items
# alone in a bifactor model to compute indices.
# This aligns with the manuscript: "The omegaH and ECV values apply
# strictly to the 26 reflective Process and Outcome items."

cat("=== Bifactor Indices (26 Reflective Items Only: Q10-Q35) ===\n")

model_bifactor_only <- '
  gen =~ Q10 + Q11 + Q12 + Q13 +
         Q14 + Q15 + Q16 + Q17 + Q18 + Q19 + Q20 +
         Q21 + Q22 + Q23 + Q24 +
         Q25 + Q26 + Q27 +
         Q28 + Q29 + Q30 +
         Q31 + Q32 + Q33 + Q34 + Q35

  assess   =~ Q10 + Q11 + Q12 + Q13
  practice =~ Q14 + Q15 + Q16 + Q17 + Q18 + Q19 + Q20
  educ     =~ Q21 + Q22 + Q23 + Q24
  follow   =~ Q25 + Q26 + Q27
  nurse_out=~ Q28 + Q29 + Q30
  patient_out=~ Q31 + Q32 + Q33 + Q34 + Q35

  gen ~~ 0*assess + 0*practice + 0*educ + 0*follow + 0*nurse_out + 0*patient_out
  assess ~~ 0*practice + 0*educ + 0*follow + 0*nurse_out + 0*patient_out
  practice ~~ 0*educ + 0*follow + 0*nurse_out + 0*patient_out
  educ ~~ 0*follow + 0*nurse_out + 0*patient_out
  follow ~~ 0*nurse_out + 0*patient_out
  nurse_out ~~ 0*patient_out
'

fit_bifactor <- cfa(model_bifactor_only, data = data, estimator = "MLR", std.lv = TRUE)

# Compute bifactor indices
bf_indices <- bifactorIndices(fit_bifactor)

# Extract key indices
evc <- bf_indices$ECV
cat(sprintf("Explained Common Variance (ECV) = %.3f\n", evc))
cat(sprintf("  Interpretation: "))
if (evc >= 0.70) cat("ECV >= 0.70 indicates essential unidimensionality.\n")

omega_h <- bf_indices$Omega_H
cat(sprintf("\nOmega Hierarchical (omegaH) = %.3f\n", omega_h))
cat(sprintf("  Interpretation: "))
if (omega_h >= 0.80) cat("omegaH >= 0.80 supports reliable total score interpretation.\n")

cat("\nOmega Hierarchical Subscale (omegaHS):\n")
omega_hs_vals <- bf_indices$Omega_HS
for (fac in names(omega_hs_vals)) {
  cat(sprintf("  %s: %.2f", fac, omega_hs_vals[[fac]]))
  if (omega_hs_vals[[fac]] < 0.50) {
    cat("  [Below 0.50 threshold; not independently reliable]\n")
  } else {
    cat("  [Above 0.50 threshold]\n")
  }
}

cat("\n")

# ------------------------------------------------------------
# 9. Formative Structural Dimension Results
# ------------------------------------------------------------
cat("=== Formative Structural Dimension (Regression on General Factor) ===\n")
reg_table <- pe[pe$op == "~" & pe$lhs == "gen", ]
reg_out <- data.frame(
  Composite = reg_table$rhs,
  Std_Beta = reg_table$std.all,
  Robust_SE = reg_table$se,
  z_value = reg_table$z,
  p_value = ifelse(reg_table$pvalue < 0.001, "<.001", sprintf("%.3f", reg_table$pvalue))
)
print(reg_out, row.names = FALSE)
cat("\n")

# ------------------------------------------------------------
# 10. Results Export
# ------------------------------------------------------------
out_dir <- "D:/Documents/Desktop/Bifactor/output"
if (!dir.exists(out_dir)) dir.create(out_dir, recursive = TRUE)

cat(sprintf("Saving results to: %s\n\n", out_dir))

# Save Table S4: Standardized loadings + Robust SE + 95% CI
write.csv(loadings_out, file = file.path(out_dir, "Table_S4_Standardized_Loadings_MLR.csv"),
          row.names = FALSE)

# Save fit indices
write.csv(fit_summary, file = file.path(out_dir, "Table_Model_Fit_Indices.csv"),
          row.names = FALSE)

# Save Bifactor Indices
bf_out <- data.frame(
  Index = c("ECV", "omegaH", names(omega_hs_vals)),
  Value = c(evc, omega_h, unlist(omega_hs_vals))
)
write.csv(bf_out, file = file.path(out_dir, "Table_Bifactor_Indices.csv"),
          row.names = FALSE)

# Save Formative Regression results
write.csv(reg_out, file = file.path(out_dir, "Table_Formative_Regression.csv"),
          row.names = FALSE)

# Save full parameter estimates (including unstandardized estimates)
write.csv(pe, file = file.path(out_dir, "Full_Parameter_Estimates_MLR.csv"),
          row.names = FALSE)

# Save lavaan summary text
sink(file = file.path(out_dir, "Lavaan_Summary_MLR.txt"))
summary(fit_mlr, standardized = TRUE, fit.measures = TRUE, ci = TRUE)
sink()

cat("=== All results saved ===\n")
cat("Files generated:\n")
cat("  1. Table_S4_Standardized_Loadings_MLR.csv\n")
cat("  2. Table_Model_Fit_Indices.csv\n")
cat("  3. Table_Bifactor_Indices.csv\n")
cat("  4. Table_Formative_Regression.csv\n")
cat("  5. Full_Parameter_Estimates_MLR.csv\n")
cat("  6. Lavaan_Summary_MLR.txt\n")
cat("\nAnalysis complete.\n")

# ============================================================
# END OF SCRIPT
# ============================================================
```

# Section 2. Full lavaan Output for the Primary MLR Model (Lavaan_Summary.txt)

Full lavaan output from the primary MLR model fit (summary with standardized = TRUE, fit.measures = TRUE, ci = TRUE). The output contains standardized factor loadings for the general and six orthogonal specific factors, robust standard errors (Huber–White sandwich estimator), z-values, p-values, residual variances, factor covariances, and the formative structural regression results.

```
lavaan 0.6-21 ended normally after 169 iterations

  Estimator                                         ML
  Optimization method                           NLMINB
  Number of model parameters                       107

  Number of observations                           410
  Number of missing patterns                         1

Model Test User Model:
                                              Standard      Scaled
  Test Statistic                              1035.765     699.111
  Degrees of freedom                               348         348
  P-value (Chi-square)                           0.000       0.000
  Scaling correction factor                                  1.482
    Yuan-Bentler correction (Mplus variant)

Model Test Baseline Model:

  Test statistic                             14176.427    8781.088
  Degrees of freedom                               403         403
  P-value                                        0.000       0.000
  Scaling correction factor                                  1.614

User Model versus Baseline Model:

  Comparative Fit Index (CFI)                    0.950       0.958
  Tucker-Lewis Index (TLI)                       0.942       0.951

  Robust Comparative Fit Index (CFI)                         0.963
  Robust Tucker-Lewis Index (TLI)                            0.957

Loglikelihood and Information Criteria:

  Loglikelihood user model (H0)              -6296.085   -6296.085
  Scaling correction factor                                  1.673
      for the MLR correction
  Loglikelihood unrestricted model (H1)      -5778.203   -5778.203
  Scaling correction factor                                  1.527
      for the MLR correction

  Akaike (AIC)                               12806.171   12806.171
  Bayesian (BIC)                             13235.899   13235.899
  Sample-size adjusted Bayesian (SABIC)      12896.368   12896.368

Root Mean Square Error of Approximation:

  RMSEA                                          0.069       0.050
  90 Percent confidence interval - lower         0.065       0.045
  90 Percent confidence interval - upper         0.074       0.054
  P-value H_0: RMSEA <= 0.050                    0.000       0.553
  P-value H_0: RMSEA >= 0.080                    0.000       0.000

  Robust RMSEA                                               0.059
  90 Percent confidence interval - lower                     0.053
  90 Percent confidence interval - upper                     0.066
  P-value H_0: Robust RMSEA <= 0.050                         0.011
  P-value H_0: Robust RMSEA >= 0.080                         0.000

Standardized Root Mean Square Residual:

  SRMR                                           0.027       0.027

Parameter Estimates:

  Standard errors                             Sandwich
  Information bread                           Observed
  Observed information based on                Hessian

Latent Variables:
                   Estimate  Std.Err  z-value
  gen =~
    Q10               0.371    0.024   15.625
    Q11               0.345    0.022   15.485
    Q12               0.400    0.024   16.967
    Q13               0.373    0.021   17.996
    Q14               0.371    0.021   17.798
    Q15               0.354    0.021   16.483
    Q16               0.393    0.023   16.934
    Q17               0.396    0.023   16.974
    Q18               0.421    0.024   17.748
    Q19               0.390    0.023   17.023
    Q20               0.457    0.025   18.466
    Q21               0.427    0.024   17.689
    Q22               0.436    0.024   18.156
    Q23               0.432    0.023   18.469
    Q24               0.453    0.024   18.667
    Q25               0.459    0.026   17.831
    Q26               0.481    0.026   18.346
    Q27               0.464    0.027   17.455
    Q28               0.419    0.023   18.505
    Q29               0.447    0.024   18.510
    Q30               0.434    0.024   18.269
    Q31               0.369    0.023   15.911
    Q32               0.392    0.022   17.525
    Q33               0.406    0.022   18.131
    Q34               0.367    0.022   16.409
    Q35               0.414    0.022   19.212
  assess =~
    Q10               0.235    0.041    5.767
    Q11               0.489    0.056    8.694
    Q12               0.344    0.047    7.364
    Q13               0.136    0.038    3.599
  practice =~
    Q14               0.197    0.047    4.202
    Q15               0.277    0.041    6.761
    Q16               0.275    0.046    5.949
    Q17               0.334    0.032   10.411
    Q18               0.324    0.038    8.470
    Q19               0.245    0.043    5.684
    Q20               0.113    0.039    2.928
  educ =~
    Q21               0.121    0.063    1.926
    Q22               0.166    0.060    2.745
    Q23               0.253    0.062    4.046
    Q24               0.141    0.044    3.218
  follow =~
    Q25               0.298    0.079    3.792
    Q26               0.301    0.077    3.899
    Q27               0.113    0.049    2.310
  nurse_out =~
    Q28               0.232    0.052    4.417
    Q29               0.299    0.043    6.953
    Q30               0.268    0.050    5.404
  patient_out =~
    Q31               0.189    0.042    4.468
    Q32               0.269    0.039    6.933
    Q33               0.244    0.037    6.567
    Q34               0.281    0.050    5.588
    Q35               0.234    0.031    7.472
  P(>|z|) ci.lower ci.upper   Std.lv  Std.all

    0.000    0.325    0.418    0.607    0.744
    0.000    0.301    0.388    0.563    0.695
    0.000    0.354    0.446    0.653    0.745
    0.000    0.333    0.414    0.610    0.804
    0.000    0.330    0.412    0.607    0.776
    0.000    0.312    0.396    0.578    0.782
    0.000    0.348    0.439    0.642    0.810
    0.000    0.351    0.442    0.648    0.839
    0.000    0.374    0.467    0.687    0.842
    0.000    0.345    0.435    0.637    0.818
    0.000    0.409    0.506    0.747    0.868
    0.000    0.380    0.474    0.698    0.854
    0.000    0.389    0.483    0.712    0.865
    0.000    0.386    0.477    0.705    0.886
    0.000    0.405    0.500    0.739    0.880
    0.000    0.408    0.509    0.749    0.858
    0.000    0.429    0.532    0.786    0.876
    0.000    0.412    0.516    0.758    0.842
    0.000    0.375    0.463    0.684    0.849
    0.000    0.399    0.494    0.730    0.874
    0.000    0.388    0.481    0.709    0.871
    0.000    0.323    0.414    0.602    0.786
    0.000    0.348    0.436    0.640    0.835
    0.000    0.362    0.450    0.663    0.854
    0.000    0.323    0.411    0.600    0.773
    0.000    0.372    0.456    0.676    0.873

    0.000    0.155    0.316    0.235    0.289
    0.000    0.378    0.599    0.489    0.604
    0.000    0.252    0.435    0.344    0.392
    0.000    0.062    0.211    0.136    0.180

    0.000    0.105    0.288    0.197    0.252
    0.000    0.197    0.358    0.277    0.375
    0.000    0.184    0.365    0.275    0.346
    0.000    0.271    0.396    0.334    0.432
    0.000    0.249    0.399    0.324    0.397
    0.000    0.160    0.329    0.245    0.315
    0.003    0.037    0.189    0.113    0.131

    0.054   -0.002    0.245    0.121    0.148
    0.006    0.047    0.284    0.166    0.201
    0.000    0.130    0.375    0.253    0.317
    0.001    0.055    0.227    0.141    0.168

    0.000    0.144    0.452    0.298    0.341
    0.000    0.150    0.452    0.301    0.336
    0.021    0.017    0.209    0.113    0.126

    0.000    0.129    0.334    0.232    0.287
    0.000    0.214    0.383    0.299    0.358
    0.000    0.171    0.365    0.268    0.329

    0.000    0.106    0.272    0.189    0.247
    0.000    0.193    0.345    0.269    0.351
    0.000    0.171    0.316    0.244    0.314
    0.000    0.183    0.380    0.281    0.363
    0.000    0.172    0.295    0.234    0.302

Regressions:
                   Estimate  Std.Err  z-value
  gen ~
    HR                0.593    0.106    5.592
    FE                0.422    0.103    4.118
    MS                0.917    0.111    8.222
  P(>|z|) ci.lower ci.upper   Std.lv  Std.all

    0.000    0.385    0.800    0.363    0.251
    0.000    0.221    0.623    0.258    0.208
    0.000    0.698    1.135    0.561    0.454

Covariances:
                   Estimate  Std.Err  z-value
 .gen ~~
    assess            0.000
    practice          0.000
    educ              0.000
    follow            0.000
    nurse_out         0.000
    patient_out       0.000
  assess ~~
    practice          0.000
    educ              0.000
    follow            0.000
    nurse_out         0.000
    patient_out       0.000
  practice ~~
    educ              0.000
    follow            0.000
    nurse_out         0.000
    patient_out       0.000
  educ ~~
    follow            0.000
    nurse_out         0.000
    patient_out       0.000
  follow ~~
    nurse_out         0.000
    patient_out       0.000
  nurse_out ~~
    patient_out       0.000
  P(>|z|) ci.lower ci.upper   Std.lv  Std.all

             0.000    0.000    0.000    0.000
             0.000    0.000    0.000    0.000
             0.000    0.000    0.000    0.000
             0.000    0.000    0.000    0.000
             0.000    0.000    0.000    0.000
             0.000    0.000    0.000    0.000

             0.000    0.000    0.000    0.000
             0.000    0.000    0.000    0.000
             0.000    0.000    0.000    0.000
             0.000    0.000    0.000    0.000
             0.000    0.000    0.000    0.000

             0.000    0.000    0.000    0.000
             0.000    0.000    0.000    0.000
             0.000    0.000    0.000    0.000
             0.000    0.000    0.000    0.000

             0.000    0.000    0.000    0.000
             0.000    0.000    0.000    0.000
             0.000    0.000    0.000    0.000

             0.000    0.000    0.000    0.000
             0.000    0.000    0.000    0.000

             0.000    0.000    0.000    0.000

Intercepts:
                   Estimate  Std.Err  z-value
   .Q10               0.454    0.099    4.605
   .Q11               0.577    0.094    6.156
   .Q12               0.544    0.101    5.403
   .Q13               0.617    0.100    6.199
   .Q14               0.369    0.093    3.971
   .Q15               0.425    0.086    4.970
   .Q16               0.317    0.087    3.631
   .Q17               0.280    0.083    3.374
   .Q18               0.208    0.087    2.373
   .Q19               0.291    0.088    3.319
   .Q20               0.143    0.089    1.607
   .Q21               0.173    0.079    2.177
   .Q22               0.352    0.091    3.879
   .Q23               0.207    0.080    2.583
   .Q24               0.123    0.085    1.455
   .Q25               0.091    0.091    1.001
   .Q26               0.048    0.091    0.529
   .Q27               0.262    0.100    2.627
   .Q28               0.295    0.082    3.590
   .Q29               0.110    0.082    1.338
   .Q30               0.186    0.083    2.253
   .Q31               0.293    0.073    4.009
   .Q32               0.359    0.083    4.321
   .Q33               0.315    0.087    3.615
   .Q34               0.397    0.087    4.546
   .Q35               0.262    0.083    3.164
  P(>|z|) ci.lower ci.upper   Std.lv  Std.all
    0.000    0.261    0.648    0.454    0.557
    0.000    0.394    0.761    0.577    0.713
    0.000    0.347    0.741    0.544    0.621
    0.000    0.422    0.812    0.617    0.814
    0.000    0.187    0.551    0.369    0.472
    0.000    0.258    0.593    0.425    0.575
    0.000    0.146    0.487    0.317    0.399
    0.001    0.117    0.443    0.280    0.363
    0.018    0.036    0.379    0.208    0.254
    0.001    0.119    0.463    0.291    0.374
    0.108   -0.031    0.317    0.143    0.166
    0.029    0.017    0.328    0.173    0.212
    0.000    0.174    0.531    0.352    0.428
    0.010    0.050    0.364    0.207    0.260
    0.146   -0.043    0.290    0.123    0.147
    0.317   -0.087    0.268    0.091    0.104
    0.597   -0.131    0.227    0.048    0.054
    0.009    0.067    0.458    0.262    0.291
    0.000    0.134    0.456    0.295    0.366
    0.181   -0.051    0.270    0.110    0.131
    0.024    0.024    0.349    0.186    0.229
    0.000    0.150    0.436    0.293    0.383
    0.000    0.196    0.521    0.359    0.468
    0.000    0.144    0.486    0.315    0.406
    0.000    0.226    0.568    0.397    0.512
    0.002    0.100    0.425    0.262    0.339

Variances:
                   Estimate  Std.Err  z-value
   .Q10               0.242    0.025    9.648
   .Q11               0.100    0.044    2.276
   .Q12               0.224    0.027    8.159
   .Q13               0.185    0.017   11.000
   .Q14               0.204    0.022    9.229
   .Q15               0.136    0.018    7.708
   .Q16               0.141    0.020    6.880
   .Q17               0.065    0.013    4.865
   .Q18               0.089    0.014    6.316
   .Q19               0.140    0.018    7.760
   .Q20               0.170    0.020    8.404
   .Q21               0.166    0.019    8.962
   .Q22               0.143    0.030    4.800
   .Q23               0.072    0.033    2.155
   .Q24               0.139    0.016    8.481
   .Q25               0.112    0.043    2.624
   .Q26               0.096    0.043    2.243
   .Q27               0.224    0.027    8.273
   .Q28               0.128    0.021    6.207
   .Q29               0.076    0.018    4.141
   .Q30               0.088    0.019    4.687
   .Q31               0.189    0.021    8.941
   .Q32               0.106    0.014    7.354
   .Q33               0.105    0.017    6.319
   .Q34               0.163    0.024    6.668
   .Q35               0.088    0.013    7.029
   .gen               1.000
    assess            1.000
    practice          1.000
    educ              1.000
    follow            1.000
    nurse_out         1.000
    patient_out       1.000
  P(>|z|) ci.lower ci.upper   Std.lv  Std.all
    0.000    0.193    0.291    0.242    0.363
    0.023    0.014    0.185    0.100    0.152
    0.000    0.170    0.277    0.224    0.291
    0.000    0.152    0.218    0.185    0.321
    0.000    0.161    0.247    0.204    0.334
    0.000    0.101    0.170    0.136    0.248
    0.000    0.101    0.181    0.141    0.224
    0.000    0.039    0.091    0.065    0.109
    0.000    0.061    0.116    0.089    0.133
    0.000    0.105    0.176    0.140    0.232
    0.000    0.130    0.210    0.170    0.230
    0.000    0.130    0.203    0.166    0.249
    0.000    0.085    0.201    0.143    0.211
    0.031    0.007    0.138    0.072    0.114
    0.000    0.107    0.172    0.139    0.198
    0.009    0.028    0.196    0.112    0.147
    0.025    0.012    0.180    0.096    0.119
    0.000    0.171    0.277    0.224    0.276
    0.000    0.087    0.168    0.128    0.196
    0.000    0.040    0.112    0.076    0.109
    0.000    0.051    0.125    0.088    0.133
    0.000    0.147    0.230    0.189    0.321
    0.000    0.077    0.134    0.106    0.180
    0.000    0.072    0.137    0.105    0.173
    0.000    0.115    0.211    0.163    0.271
    0.000    0.063    0.113    0.088    0.147
             1.000    1.000    0.375    0.375
             1.000    1.000    1.000    1.000
             1.000    1.000    1.000    1.000
             1.000    1.000    1.000    1.000
             1.000    1.000    1.000    1.000
             1.000    1.000    1.000    1.000
             1.000    1.000    1.000    1.000
```

# Section 3. Result Tables

**Table 3.1. MLR-scaled model fit indices for the primary bifactor model (N = 410).**

| **Index** | **Value** |
| --- | --- |
| χ²(scaled) | 699.11 |
| df | 348 |
| p-value | 0.000 |
| CFI | 0.958 |
| TLI | 0.951 |
| RMSEA | 0.050 |
| RMSEA_Lower | 0.045 |
| RMSEA_Upper | 0.054 |
| SRMR | 0.027 |

**Table 3.2. Bifactor indices computed on the 26 reflective items only (pure measurement model, excluding the formative structural composites).**

| **Index** | **Value** | **Manuscript_Reported** | **Threshold** | **Interpretation** |
| --- | --- | --- | --- | --- |
| ECV (Explained Common Variance) | 0.868608 | 0.869 | >= 0.70 | Essential unidimensionality met |
| PUC (Proportion of Uncontaminated Correlations) | 0.849231 | — | >= 0.70 | Supporting unidimensionality met |
| Omega (Total reliability) | 0.988546 | — | — | Total reliability of general factor |
| OmegaH (Hierarchical Omega) | 0.963148 | 0.963 | >= 0.80 | Reliable total score interpretation supported |
| OmegaHS_assess (Assessment subscale) | 0.187696 | 0.19 | >= 0.50 | Not independently reliable (below threshold) |
| OmegaHS_practice (Practice subscale) | 0.133289 | 0.13 | >= 0.50 | Not independently reliable (below threshold) |
| OmegaHS_educ (Education subscale) | 0.051410 | 0.05 | >= 0.50 | Not independently reliable (below threshold) |
| OmegaHS_follow (Follow-up subscale) | 0.078001 | 0.08 | >= 0.50 | Not independently reliable (below threshold) |
| OmegaHS_nurse_out (Nurse Outcomes subscale) | 0.109431 | 0.11 | >= 0.50 | Not independently reliable (below threshold) |
| OmegaHS_patient_out (Patient Outcomes subscale) | 0.117785 | 0.12 | >= 0.50 | Not independently reliable (below threshold) |

**Table 3.3. Standardized regression weights of the three formative structural composites (Human Resources, Facilities & Equipment, Management Systems) on the general factor, with robust SEs, z-values, and p-values.**

| **Composite** | **Std_Beta** | **Robust_SE** | **z_value** | **p_value** |
| --- | --- | --- | --- | --- |
| HR | 0.250714733420859 | 0.10599909661175 | 5.59161824114349 | <.001 |
| FE | 0.208080031147283 | 0.102522801272589 | 4.11806370521496 | <.001 |
| MS | 0.453738322840354 | 0.11147086832007 | 8.22223301869672 | <.001 |

**Table 3.4. Standardized factor loadings (Std_Loading) for the general factor (gen) and the six orthogonal specific factors, with MLR robust standard errors, z-values, p-values, and 95% Wald confidence intervals for the unstandardized estimates (CI_95_Lower / CI_95_Upper). Note: the 95% CI columns report Wald intervals for unstandardized estimates; standardized loading confidence intervals would require the delta method or bootstrap computation, which was precluded by pervasive convergence failures in the orthogonal bifactor resampling.**

| **Factor** | **Item** | **Std_Loading** | **Robust_SE** | **z_value** | **p_value** | **CI_95_Lower** | **CI_95_Upper** |
| --- | --- | --- | --- | --- | --- | --- | --- |
| gen | Q10 | 0.744 | 0.024 | 15.625 | <.001 | 0.325 | 0.418 |
| gen | Q11 | 0.695 | 0.022 | 15.485 | <.001 | 0.301 | 0.388 |
| gen | Q12 | 0.745 | 0.024 | 16.967 | <.001 | 0.354 | 0.446 |
| gen | Q13 | 0.804 | 0.021 | 17.996 | <.001 | 0.333 | 0.414 |
| gen | Q14 | 0.776 | 0.021 | 17.798 | <.001 | 0.330 | 0.412 |
| gen | Q15 | 0.782 | 0.021 | 16.483 | <.001 | 0.312 | 0.396 |
| gen | Q16 | 0.810 | 0.023 | 16.934 | <.001 | 0.348 | 0.439 |
| gen | Q17 | 0.839 | 0.023 | 16.974 | <.001 | 0.351 | 0.442 |
| gen | Q18 | 0.842 | 0.024 | 17.748 | <.001 | 0.374 | 0.467 |
| gen | Q19 | 0.818 | 0.023 | 17.023 | <.001 | 0.345 | 0.435 |
| gen | Q20 | 0.868 | 0.025 | 18.466 | <.001 | 0.409 | 0.506 |
| gen | Q21 | 0.854 | 0.024 | 17.689 | <.001 | 0.380 | 0.474 |
| gen | Q22 | 0.865 | 0.024 | 18.156 | <.001 | 0.389 | 0.483 |
| gen | Q23 | 0.886 | 0.023 | 18.469 | <.001 | 0.386 | 0.477 |
| gen | Q24 | 0.880 | 0.024 | 18.667 | <.001 | 0.405 | 0.500 |
| gen | Q25 | 0.858 | 0.026 | 17.831 | <.001 | 0.408 | 0.509 |
| gen | Q26 | 0.876 | 0.026 | 18.346 | <.001 | 0.429 | 0.532 |
| gen | Q27 | 0.842 | 0.027 | 17.455 | <.001 | 0.412 | 0.516 |
| gen | Q28 | 0.849 | 0.023 | 18.505 | <.001 | 0.375 | 0.463 |
| gen | Q29 | 0.874 | 0.024 | 18.510 | <.001 | 0.399 | 0.494 |
| gen | Q30 | 0.871 | 0.024 | 18.269 | <.001 | 0.388 | 0.481 |
| gen | Q31 | 0.786 | 0.023 | 15.911 | <.001 | 0.323 | 0.414 |
| gen | Q32 | 0.835 | 0.022 | 17.525 | <.001 | 0.348 | 0.436 |
| gen | Q33 | 0.854 | 0.022 | 18.131 | <.001 | 0.362 | 0.450 |
| gen | Q34 | 0.773 | 0.022 | 16.409 | <.001 | 0.323 | 0.411 |
| gen | Q35 | 0.873 | 0.022 | 19.212 | <.001 | 0.372 | 0.456 |
| assess | Q10 | 0.289 | 0.041 | 5.767 | <.001 | 0.155 | 0.316 |
| assess | Q11 | 0.604 | 0.056 | 8.694 | <.001 | 0.378 | 0.599 |
| assess | Q12 | 0.392 | 0.047 | 7.364 | <.001 | 0.252 | 0.435 |
| assess | Q13 | 0.180 | 0.038 | 3.599 | <.001 | 0.062 | 0.211 |
| practice | Q14 | 0.252 | 0.047 | 4.202 | <.001 | 0.105 | 0.288 |
| practice | Q15 | 0.375 | 0.041 | 6.761 | <.001 | 0.197 | 0.358 |
| practice | Q16 | 0.346 | 0.046 | 5.949 | <.001 | 0.184 | 0.365 |
| practice | Q17 | 0.432 | 0.032 | 10.411 | <.001 | 0.271 | 0.396 |
| practice | Q18 | 0.397 | 0.038 | 8.470 | <.001 | 0.249 | 0.399 |
| practice | Q19 | 0.315 | 0.043 | 5.684 | <.001 | 0.160 | 0.329 |
| practice | Q20 | 0.131 | 0.039 | 2.928 | 0.003 | 0.037 | 0.189 |
| educ | Q21 | 0.148 | 0.063 | 1.926 | 0.054 | -0.002 | 0.245 |
| educ | Q22 | 0.201 | 0.060 | 2.745 | 0.006 | 0.047 | 0.284 |
| educ | Q23 | 0.317 | 0.062 | 4.046 | <.001 | 0.130 | 0.375 |
| educ | Q24 | 0.168 | 0.044 | 3.218 | 0.001 | 0.055 | 0.227 |
| follow | Q25 | 0.341 | 0.079 | 3.792 | <.001 | 0.144 | 0.452 |
| follow | Q26 | 0.336 | 0.077 | 3.899 | <.001 | 0.150 | 0.452 |
| follow | Q27 | 0.126 | 0.049 | 2.310 | 0.021 | 0.017 | 0.209 |
| nurse_out | Q28 | 0.287 | 0.052 | 4.417 | <.001 | 0.129 | 0.334 |
| nurse_out | Q29 | 0.358 | 0.043 | 6.953 | <.001 | 0.214 | 0.383 |
| nurse_out | Q30 | 0.329 | 0.050 | 5.404 | <.001 | 0.171 | 0.365 |
| patient_out | Q31 | 0.247 | 0.042 | 4.468 | <.001 | 0.106 | 0.272 |
| patient_out | Q32 | 0.351 | 0.039 | 6.933 | <.001 | 0.193 | 0.345 |
| patient_out | Q33 | 0.314 | 0.037 | 6.567 | <.001 | 0.171 | 0.316 |
| patient_out | Q34 | 0.363 | 0.050 | 5.588 | <.001 | 0.183 | 0.380 |
| patient_out | Q35 | 0.302 | 0.031 | 7.472 | <.001 | 0.172 | 0.295 |

# Key Technical Notes

**Bootstrap failure.** The Bollen–Stine bootstrap (2000 samples) was attempted but produced pervasive convergence failures and Heywood cases in the orthogonal bifactor resampling. Consequently, all inferential statistics reported in the manuscript rely on MLR robust standard errors (Huber–White sandwich estimator), which provide consistent parameter estimates under multivariate non-normality when bootstrap resampling is unavailable (Kline, 2016).

**Bifactor indices computation.** The BifactorIndicesCalculator package requires models without exogenous predictors. Therefore, ECV, ωH, and ωHS were computed on a pure measurement model of the 26 reflective items only, consistent with the manuscript specification that these indices apply strictly to the reflective core (Table 2 note).

**Data availability.** The raw data file (data410.csv) is not included in this supplementary file due to participant confidentiality. Researchers wishing to reproduce the analysis should format their data with 35 columns named Q1 through Q35, containing integer values from 1 to 5 on a Likert scale.

# Correspondence with the Manuscript

| **File / Table in this document** | **Corresponding manuscript content** |
| --- | --- |
| Section 1 (R syntax) + Section 2 (lavaan output) | Reproducible syntax and full output for the bifactor model (manuscript Methods, Section 2.3.5) |
| Table 3.4 (Table_S4_Loadings_MLR.csv) | Standardized loadings reported in the manuscript (Methods, Section 2.3.5; Supplementary File 2, Table S4) |
| Table 3.2 (Table_Bifactor_Indices.csv) | Bifactor indices reported in manuscript Table 2 (ECV = 0.869; ωH = 0.963; ωHS = 0.05–0.19) |
| Table 3.1 (Table_Fit_Indices.csv) | Model fit indices reported in manuscript Table 3 (primary bifactor model) |
| Table 3.3 (Table_Formative_Regression.csv) | Formative regression weights reported in manuscript Results, Section 3.2.4 |
